# Modeling co-occupancy of transcription factors using chromatin features

**DOI:** 10.1093/nar/gkv1281

**Published:** 2015-11-20

**Authors:** Liang Liu, Weiling Zhao, Xiaobo Zhou

**Affiliations:** Center for Bioinformatics and Systems Biology, Department of Radiology, Wake Forest School of Medicine, Winston-Salem, NC 27157, USA

## Abstract

Regulation of gene expression requires both transcription factor (TFs) and epigenetic modifications, and interplays between the two types of factors have been discovered. However study of relationships between chromatin features and TF–TF co-occupancy remains limited. Here, we revealed the relationship by first illustrating distinct profile patterns of chromatin features related to different binding events, including single TF binding and TF–TF co-occupancy of 71 TFs from five human cell lines. We further implemented statistical analyses to demonstrate the relationship by accurately predicting co-occupancy genome-widely using chromatin features including DNase I hypersensitivity, 11 histone modifications (HMs) and GC content. Remarkably, our results showed that the combination of chromatin features enables accurate predictions across the five cells. For individual chromatin features, DNase I enables high and consistent predictions. H3K27ac, H3K4me 2, H3K4me3 and H3K9ac are more reliable predictors than other HMs. Although the combination of 11 HMs achieves accurate predictions, their predictive ability varies considerably when a model obtained from one cell is applied to others, indicating relationship between HMs and TF–TF co-occupancy is cell type dependent. GC content is not a reliable predictor, but the addition of GC content to any other features enhances their predictive ability. Together, our results elucidate a strong relationship between TF–TF co-occupancy and chromatin features.

## INTRODUCTION

Transcriptional regulation exists at both genetic and epigenetic levels. Binding of transcription factors (TFs) to specific DNA sequences is a pivotal step in the control of gene expression. Studies of sequence-associated TF binding preferences have led to the development of sequence-specific Position Weighted Matrix (PWM) (1) and position-specific affinity matrices (2) approaches for identification of TF binding sites (TFBSs).

Epigenetic regulation refers to the alteration of DNA accessibility to TFs coordinately with chemical modifications of chromatin (3). This process may involve in multiple factors, such as DNA shape, chromatin accessibility, histone modifications (HMs), nucleosome positions and other chromatin variants (4–9). Analyses of experimental data show that distinct HM patterns appear around TFBSs, and ChIP-Seq signals of TF bindings and HMs are highly predictive of each other (10–14). Specifically, previous studies depicted that chromatin features, such as HMs and DNA shape, are highly correlated with the quantitative changes of TF binding affinities (14,15).

TFs tend to work with others for accurately regulating expression of their target genes by binding to the same regulatory regions (16). These TFs can act either collaboratively or competitively (17–20), and are tightly associated with modeling of cell-specific *cis*-regulatory modules (21,22). Experimental studies of possible TF–TF interactions with either systematic assays or ChIP-Seq in various organisms, such as *Escherichia coli*, yeast, the *Drosophila* embryo and human cell lines (17,23–27), revealed a great number of co-localization hotspots (28), and co-localization patterns that are related to regulatory functions (21). For instance, CCCTC-binding factor (CTCF) is a TF and widely binds to thousands of loci in genome (29). CTCF performs myriad functions by controlling binding affinities with its partners (30), such as yin yang 1 (YY1). The cooperative role of CTCF and YY1 was originally seen in *trans*-activating *Tsix* ncRNA during X-chromosome inactivation (31). Genome-wide analysis depicted their global co-localizations in human cells (23), and their interactions are, at least in part, associated with the evolutionary stability of CTCF genomic occupancy (19). Even for the same TF, if two binding sites are close to each other, the binding of the TF to one site is likely to interfere its binding to another one (32).

DNA sequence and chemical modifications of chromatin can affect not only binding of an individual TF but also a cluster of TFs (23,33–35). Although a large amount of works have been done in investigating the associations of chromatin features with bindings of individual TFs (10–15), a few of studies have devoted to explore the relationships between chromatin features and TF–TF co-occupancy/interactions. This may shed light on a comprehensive understanding of the relationships between TF–TF interactions and chromatin features, as well as their regulatory mechanisms.

In this work, we firstly illustrated the distinct profiling patterns of chromatin features for two types of genome binding events, including the regions solely bound by an individual TF and others bound by this TF and its partners simultaneously. We aligned and compared the profiles of DNase I hypersensitivity (DNase I), HMs and TF binding events by taking advantage of the wealth of data from the ENCODE project (23). Statistical tests showed a strong correlation between binding events and chromatin features across five human cell lines, including A549, GM12878, H1-hESC, HepG-2 and K562. To further demonstrate the relationship between binding events and chromatin features, we then examined predictive ability of chromatin features for TF–TF co-occupancy through a computational model. Our results showed that chromatin features are able to accurately predict the TF–TF co-occupancy genome-widely. By constructing computational models with different chromatin features, we found that both DNase I and combined 11 HMs achieve similar predictive powers. In general, the predictive ability of a single HM is weak; 4 out of 11 HMs, including H3K27ac, H3K4me 2, H3K4me3 and H3K9ac, are more reliable predictors than others. Although GC content itself is not an accurate predictor, addition of GC content improves the predictive ability of DNase I or HMs. We consequently applied the models obtained from one cell line to other cells, and found that the prediction accuracy of the combined chromatin features, including DNase I, 11 HMs and GC content, is maintained consistent across cell lines. Prediction accuracies of the models with individual or the combined 11 HMs receive considerable variances across cell lines, indicating the correlation between HMs and TF–TF co-occupancy is cell type dependent. Models using DNase I on the other hand obtain more consistent predictions across all of cell lines. Taken together, our analyses depict a potential role of chromatin features as determinants in the prediction of TF–TF co-occupancy. This study will contribute to our understanding of the interplay between genetic and epigenetic regulations of gene expression.

## MATERIALS AND METHODS

### Datasets

All of the data used in this study were downloaded from the ENCODE project (http://genome.ucsc.edu/ENCODE/downloads.html) (1). The ENCODE project has generated TF binding data, by using ChIP-Seq technique (2), in both normal and cancer cell lines. Five human cell lines were selected in this study, including A549 (epithelial cells), GM12878 (B-lymphoblastoid cell), H1-hESC (embryonic stem cells), HepG-2 (hepatocellular carcinoma cells) and K562 (erythrocytic leukemia cells). TF binding profiles by ChIP-Seq data were obtained from the HAIB, and UW TFBS ENCODE groups.

Genome-wide profiles of HMs, including H3K9ac, H3K27ac, H3K4me3, H3K4me2, H3K4me1, H3K79me2, H3K9me3, H3K27me3, H3K36me3 and H4K20me1, and the histone variant, H2A.z, were generated using the ChIP-Seq technique (2). DNase I profiles of the five cell lines were generate with DNase-Seq technique (3).

DNA methylation levels were quantitatively profiled with the RRBS technique and Infinium HumanMethylation450 BeadChip array. The former covers >1 M CpG sites, while the latter measures the methylation levels for 485 577 CpG sites. The methylation level of each CpG is determined as the average of RRBS replicated experiments or HumanMethylation450 BeadChip data and ∼1.3 M CpGs were included.

Genomic locations of 40 193 genes with all information were extracted from the human genome version hg19, obtained from the RefSeq database (downloaded from UCSC Genome Browser at http://genome.ucsc.edu/).

We downloaded the RNA-Seq data that were profiled using Poly A+ protocol from the ENCODE project (1). The expression levels of all RefSeq genes were calculated according to the FPKM (Fragments Per Kilobase of transcript per Million mapped reads) definition. To reduce the redundancy, the expression levels from multiple replicates were merged by taking the mean expression level of each gene.

Chromatin state segmentation data was also downloaded from the USCS Genome Browser. The chromatin states were defined using the unsupervised machine learning technique ChromHMM (36), and available for the GM12878, H1-hESC, HepG-2 and K562 cell lines.

### Determination of TF–TF co-occupying regions

Based on the uniform processing pipeline developed for the ENCODE Integrative Analysis effort (37), the binding sites or each TF were determined by peak calling using the SPP peak caller (38) and the consistency and reproducibility between biological replicates with the measurement of the Irreproducible Discovery Rate (IDR < 2%) (39), from the corresponding ChIP-Seq data. The ChIP-Seq peak summits were selected to represent TFBSs. There are various numbers of binding sites for each TF across different cell lines (Supplementary Table S1).

The BEDTools intersectBED function (40) was used to determine whether two TFs, such as CTCF and YY1, were co-localized in the same genomic regions (18–20,41,42). Here we named, for example, the genome regions co-occupied by CTCF–YY1 as CTCF–YY1 co-occupancy, if at least a 30% overlap of CTCF peak by YY1 peak, and *vice versa*, was observed in this region. In contrast, we defined genome regions bound by CTCF but not YY1 as CTCF-only events, and regions bound by YY1 not CTCF as YY1-only events. In such way, all binding sites can be classified into three binding event categories, CTCF-only, CTCF–YY1/YY1–CTCF and YY1-only (Supplementary Figure S1A). Of note, using different overlapping criteria, such as 40% overlapped ChIP-Seq peaks, or other tools, such as IntervalStats (43), only resulted in the change of numbers of TFBSs in each binding category, but not the following prediction analysis or association study (data not shown).

### Sequence and chromatin features at TFBSs

We examined the sequence features among binding sites by testing the binding motifs of each TF. Taken CTCF and YY1 as an example, for the different binding events, including CTCF-only, CTCF–YY1 and YY1-only, we analyzed DNA sequences surrounding binding sites of CTCF or YY1. We used the top 1000 binding sites in each type of binding events to identify the motifs, and discovered the *de novo* motifs using MEME tool (44).

For profiles of chromatin features including DNase I and HMs, we first selected the 6k-bp genome regions centered at peak summits of ChIP-Seq data for each TF to analyze the differences related to binding events. We calculated the profiles of tag density of chromatin features at a resolution of 100 bp, and quantified tag density in RPKM. The HM patterns at TFBSs were characterized by 11 types of histone methylation and acetylation, each of which has been associated with transcriptional activation, suppression or both (7,36,45).

We also selected the 100-bp region centered at each TFBS, and calculated the normalized RPKM values of chromatin features in this small region to represent their densities. Then Student's *t*-tests were performed between the profiles of TF–TF co-occupancy and TF-only binding sites. Similar analysis was performed to sequence features, such as GC content.

For DNA methylation, we selected the methylation level of CpG site(s) mapped into the 100-bp region to compute methylation level at this TFBS. For 100-bp regions centered at TFBSs with more than one CpG site, the average of methylation levels over these mapped CpG sites was selected to represent the methylation level.

### Predicting TF binding events

We have examined the HMs, DNase I and GC content in the 100-bp region centered at TFBSs (ChIP-Seq peak summits), by counting and normalizing the number of reads mapping to this region to calculate RPKM values. The TF–TF and TF-only binding sites (e.g. CTCF–YY1 versus CTCF-only) were selected as ‘positive’ and ‘negative’ datasets, respectively.

**Figure 1.**
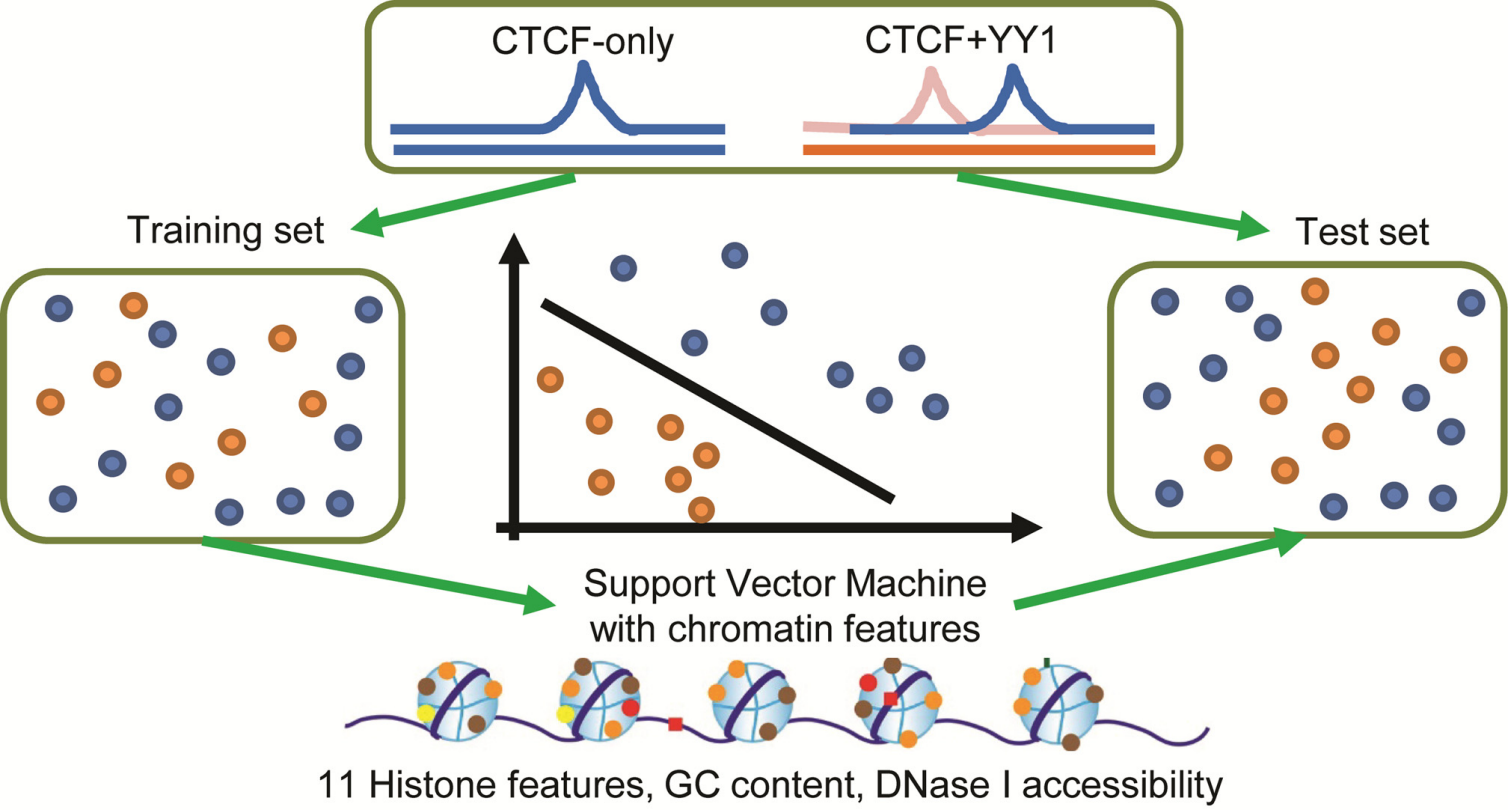
Schematic of the SVM approach used to predict TF–TF and TF-only, or TF–TF and TF-only binding events. YY1 was selected as a representation of CTCF binding partners for illustration. All binding events, including CTCF-TF co-occupying and CTCF-/TF-only binding regions, were separated into training and testing datasets. Then SVM classifier was trained using all or a subset of 11 HMs, DNase I and GC content. The trained model was applied to the test dataset for prediction accuracy valuation with ROC curves, AUC and ACC values. The model was also applied to the same TF–TF pair in different cell types for cross-cell type tests.

We used two non-linear classifiers and two linear methods to build the chromatin models for studying the correlations between TF–TF co-occupancy and chromatin features (Figure 1). The two non-linear classifiers were support vector machine (SVM) (46) and Random Forest (RF). The linear methods included Naïve Bayes (NB) and Linear Discriminant Analysis (LDA). For the SVM classifier, LIBSVM software (47) implemented in the R package ‘e1071’ and non-linear radial basis kernel were selected. R packages ‘randomForest’, ‘e1071’, and ‘MASS’ were used for the RF, NB and LDA, respectively.

In each cell line, we randomly selected two-third of positive and negative datasets as training and the rest as testing. The ability of chromatin model to distinguish TF–TF co-occupancy from TF-only binding events was assessed by examination of receiver operator characteristic (ROC) curves, plotting the true-positive rate versus the false-positive rate. To test the stability of these predictions, the above procedure was repeated 10 times and the means of the area under ROC curves (AUC) and prediction accuracy (ACC) values were computed to represent the prediction accuracy.

The learned models can be applied to different cell lines for the cross-cell type testing purpose. During this process, the model learned for one pair of TFs was used to other cells for the same TF–TF pair. The prediction accuracies were evaluated by the calculations of AUC and ACC values.

## RESULTS

### Analysis of sequence and chromatin features for individual TF binding and TF–TF co-occupying events

TFs account for ∼10% of proteins encoded by human genes (48) and their bindings are depended on both genome sequence and chemical alternatives to the sequence (10–14). Based on the assessed TFs in the ENCODE project (23), a large number of TF–TF binding partners have been identified (17,27). In this study, we used CTCF and SP1 as the key experimental TFs, and analyzed their co-occupancy with other TFs. We also selected a set of TFs, including ATF3, GABP, NRSF, POL2, USF1 and YY1, whose ChIP-Seq data were available in all of the five human cell lines, to further demonstrate the relationships between chromatin features and binding events. Of note, the analyzing approach presented in this work can be feasibly applied to other TFs.

A TF may share its binding regions with its partner, namely TF–TF co-occupancy. For instance, CTCF–YY1 co-occupancy refers to the regions co-occupied by CTCF and YY1. Consequently, we defined the genomic regions bound by CTCF but not YY1 as CTCF-only events, and the regions bound by YY1 but not CTCF as YY1-only events (Supplementary Figure S1A). For each TF–TF pair, there are various numbers of co-occupying and solely binding events (see Supplementary Materials; Supplementary Figure S1B and Supplementary Table S1). The co-occupying TFs preferably bind at specific genomic regions (Supplementary Figure S2A). Genome-wide analyses of TFBSs revealed that CTCF intends to bind at gene bodies; while its partner, YY1, as an example, prefers to regulate its target genes by binding at proximal promoters. When considering co-localized CTCF and YY1, their co-occupied regions are mainly distributed in the gene promoters (Supplementary Figure S2B). In our analysis, we considered the genome-wide TFBSs.

By aligning the profiles of DNA sequences, DNase I and HMs, we were able to examine the chromatin features for TF solely and TF–TF co-occupying binding events. *De novo* binding motifs analyses of each TF–TF pair (see ‘Materials and Methods’ section) illustrated a similar sequence preference for TFs involved in both co-occupying and solely binding regions (see Supplementary Materials; e.g. motifs of CTCF and YY1 shown in Supplementary Figure S3), indicating that DNA sequence may not be a determinant for the TF–TF co-occupancy.

**Figure 2.**
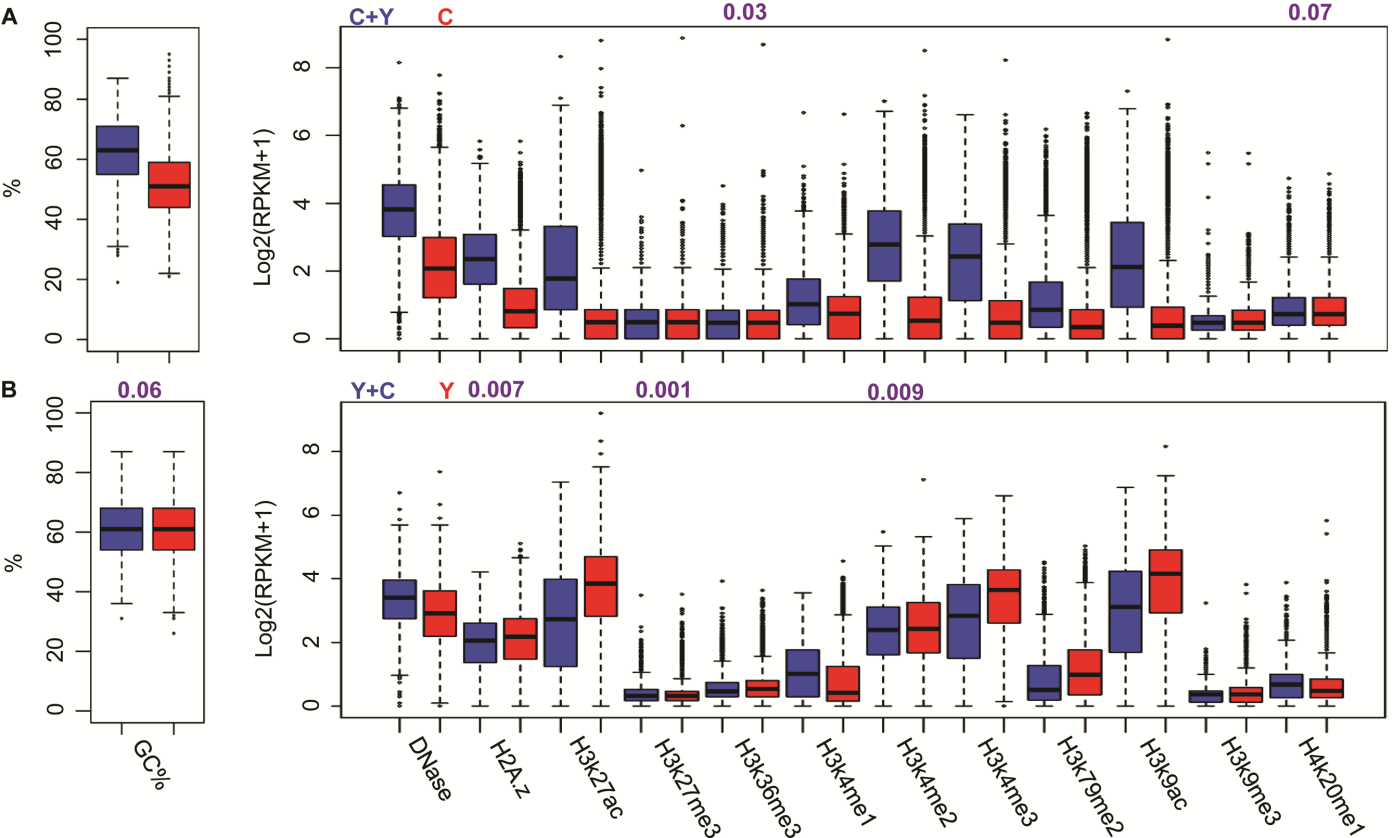
Comparison of chromatin feature profiles (**A**) between CTCF–YY1 co-occupying (C + Y, blue) and CTCF-only binding regions (C, red) and (**B**) between YY1-CTCF (Y + C, blue) and YY1-only (Y, red) binding regions in the K562 cell line. All tests reached *P*-values < 1.0E-5, unless values are shown in figures (numbers in purple).

Analyses of GC content (see ‘Materials and Methods’ section), which dictates nucleosome depletion at mammalian promoters with GC-richness benefiting TF binding (17), showed that CTCF–YY1 co-occupying regions are significantly associated with GC content than CTCF-only binding sites (Student's *t*-test *P* < 1e-15; see Supplementary Materials; Figure 2 and Supplementary Figure S4), suggesting a stronger transcriptional activities of the CTCF–YY1 co-occupied regions (19,49,50). This is consistent with previous findings that genes with CTCF–YY1 co-occupying regions are highly expressed than others solely bound by CTCF (19). It is worth to note that minor differences exist in GC-content profiles, and the chromatin feature profiles as follows, between CTCF-TF and TF-CTCF binding events, because the binding sites of co-occupied CTCF and YY1, represented by ChIP-Seq peak summits, are not located at the exactly same genome positions.

Analyses of chromatin features also revealed distinct profiling patterns for different binding events (see Supplementary Materials; Supplementary Figure S5). The HM patterns at TFBSs were characterized by 11 types of histone methylation and acetylation (see ‘Materials and Methods’ section), which are associated with transcriptional activation, suppression or both (7,36,45). DNase I hypersensitive sites are regions of chromatin sensitive to cleavage by DNase I. In these sites, nucleosome structure is less compacted, increasing the availability of the DNA to binding of TFs (35,51). Our results show that, HMs, except H3K27me3, are more enriched in CTCF–YY1 co-occupying regions than these in the CTCF-only sites, which is consistent with the reported association between co-localizations of CTCF and YY1 and transcription activity (19). When only considering a smaller 100-bp regions centered at TFBSs, the differences of chromatin feature enrichment are more obvious. All comparisons of individual chromatin features show significantly differences, with a few exceptions such as H3K27me3 in the HepG-2 cell line (see Supplementary Materials; Supplementary Figure S4).

We did the same analysis for other TF–TF pairs. Similar enrichment patterns were observed when comparing chromatin feature profiles between TF–TF co-occupancy and TF-only events (student's *t*-test *P* < 0.05; see Supplementary Materials; Figure 2, Supplementary Figure S4 and Supplementary Table S2). Taken together, our analyses suggest that chromatin features are strongly related to TF binding events, and encourage us to construct a computational model using chromatin features to discriminate TF–TF co-occupancy from TF-only events.

### Chromatin features are predictive of TF–TF co-occupancy

We used an SVM classifier to study the direct relationship between local chromatin features and TF–TF co-occupancy, by evaluating to what extent the local chromatin features are informative of a variety of binding events. The classifier was constructed based on the normalized signals (log_2_-transformed RPKMs) of chromatin features within the 100-bp window centered at TF peak summits (see ‘Materials and Methods’ section; Figure 1), and tested on its predictive ability by examination of ROC curves, together with the means of AUC and ACC values after 10-time repetition. The chromatin features includes DNase I, 11 HMs and GC content.

**Figure 3.**
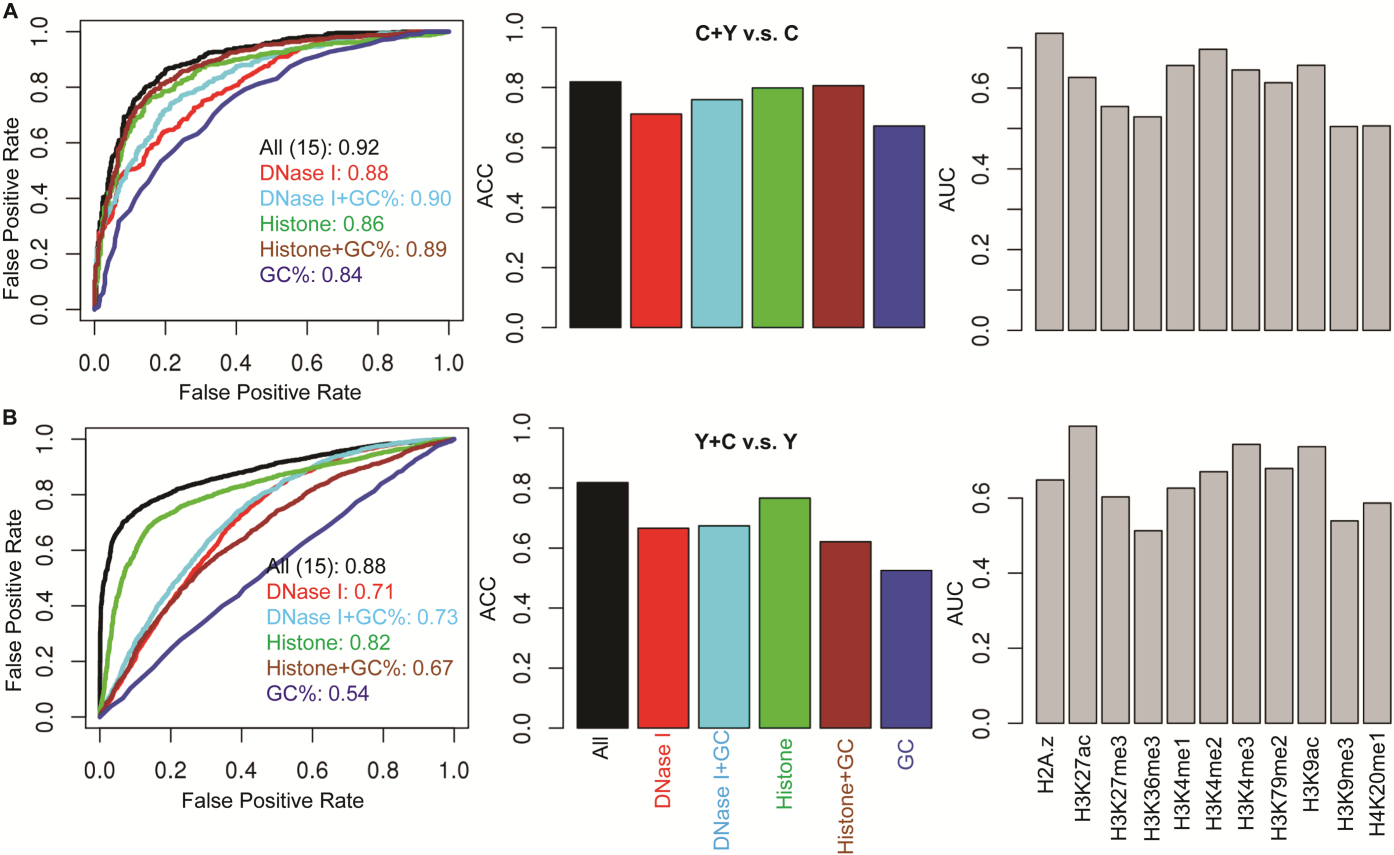
Chromatin features are predictive of CTCF–YY1 co-occupancy from (**A**) CTCF-only and (**B**) YY1-only events with high accuracies in the GM12878 cell line. Left: ROC curves are shown with colors representing predictions using different chromatin features and AUC values are indicated in the legend; Middle: predictions evaluated with ACCs; and Right: predictions evaluated with AUC values using individual histone features.

Starting with CTCF and YY1 binding events, the chromatin feature-based model enabled accurate predictions of co-occupancy, with AUC = 0.92 and 0.88 for CTCF–YY1 (distinguished from CTCF-only) and YY1–CTCF (distinguished from YY1-only) binding events, in the GM12878 cell line (Figure 3). High prediction accuracies were also achieved in the A549, H1-hESC, HepG-2 and K562 cells with AUC = 0.92 and 0.76, 0.81 and 0.85, 0.91 and 0.78, and 0.89 and 0.79, respectively (Supplementary Figure S6). The predictive ability of chromatin features were also demonstrated using ACC value estimations with an average of ACCs ∼0.80 for CTCF–YY1 and ∼0.75 for YY1–CTCF co-occupancy (Figure 3, Supplementary Figure S6 and Supplementary Table S3).

When using individual or a subset of chromatin features as predictors, the prediction accuracies obtained from DNase I and combined 11 HMs are closed to that using all features as a whole (Figure 3, Supplementary Figure S6 and Supplementary Table S3). This observation may be explained by the previously reported results indicating that both DNase I and HMs can be used to precisely estimate open chromatin (52,53) and HMs are predictive of chromatin accessibility (54).

GC content is another valuable predictor. GC content patterns are not significantly different, especially in the YY1–CTCF and YY1-only comparisons, therefore GC content itself does not enable accurate predictions. However, addition of GC content to any other features enhances their prediction capability (Figure 3, Supplementary Figure S6 and Supplementary Table S3).

We also employed the RF, NB and LDA for the prediction of CTCF–YY1 co-occupancy. High prediction accuracies were generally achieved. For instance, the RF Classifier can achieve high predictions with AUC = 0.90 and 0.76, 0.89 and 0.87, 0.79 and 0.85, 0.91 and 0.78, and 0.89 and 0.79, respectively, in the A549, GM12878, H1-hESC, HepG-2 and K562 cells, when using all chromatin features as a predictor. Similar results were observed when using different chromatin features as predictors (Supplementary Figure S7). The linear model with NB and LDA gave accurate predictions from individual chromatin features, such as DNase I, which were similar to the results obtained from non-linear models. However, the prediction accuracy from combined chromatin features, especially using 11 histone marks as a predictor, was low (Supplementary Figures S8 and S9), indicating a non-linear relationship of HMs with TF–TF co-occupancy. These results were consistent with the relationship between epigenetic modifications and individual TF binding (14). Since the SVM classifier led to better predictions, this method is selected to depict the predictive ability and consequently correlation in the following analyses.

**Figure 4.**
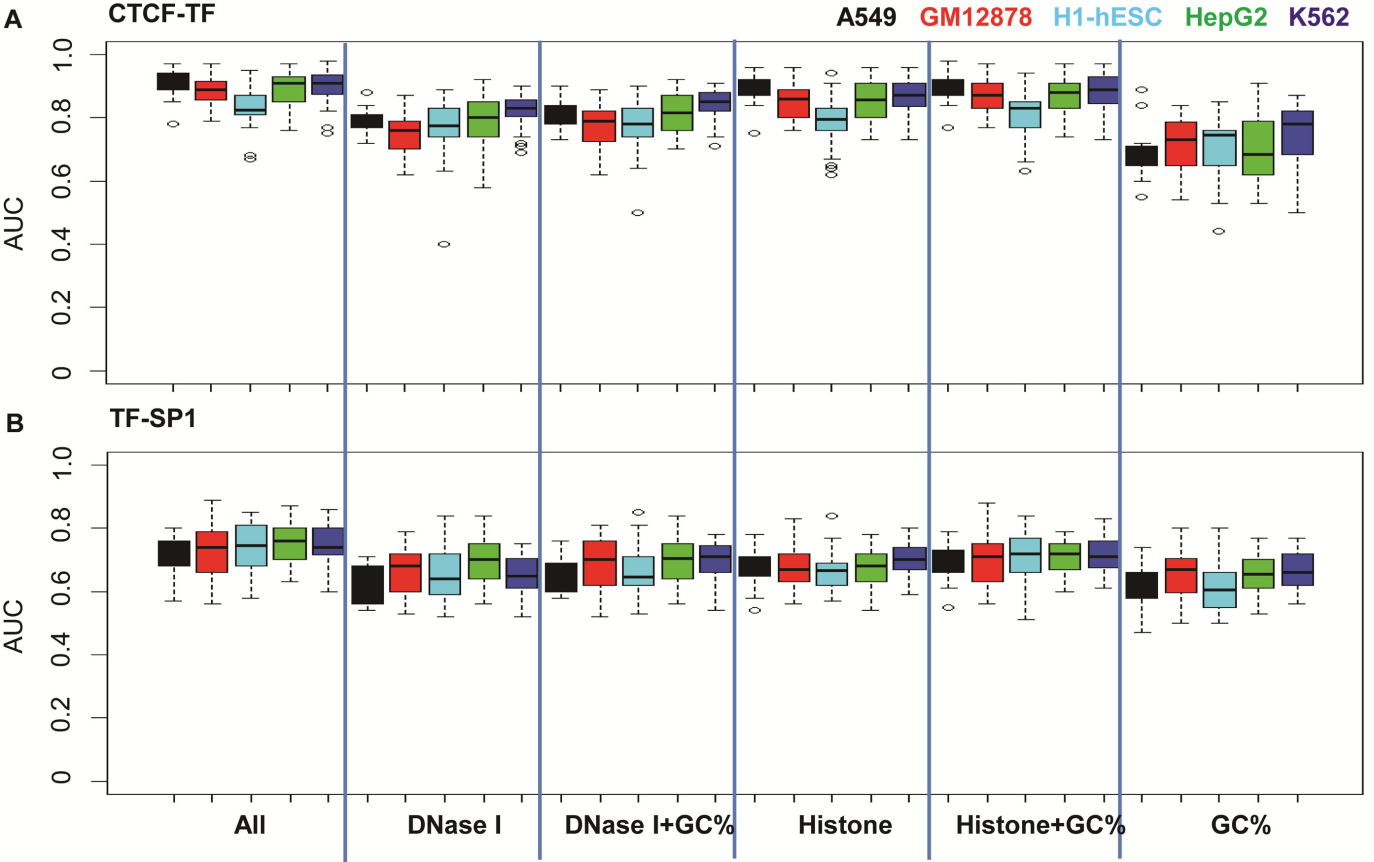
Chromatin features are predictive of (**A**) CTCF-TF and (**B**) TF-CTCF co-occupancy with high accuracies. Computational models were trained and applied to the same TF–TF pair in the same cell line, labeled by colors: black, A549; red, GM12878; cyan, H1-hESC; green, HepG-2; and blue, K562. Models were trained using different sets of chromatin features.

The SVM classifiers were trained and tested for other types of TFs for prediction of CTCF-TF/TF-CTCF co-occupancy. The results showed that, chromatin features are informative of binding events, with mean AUC values 0.90 and 0.72, 0.89 and 0.73, 0.83 and 0.73, 0.88 and 0.75, and 0.90 and 0.74 in the A549, H1-hESC, HepG-2 and K562 cells, for both CTCF–YY1 and TF-CTCF co-occupancy, respectively (Figure 4 and Supplementary Table S3). Consistent with the observations from CTCF–YY1 analysis, both the combined 11 HMs and DNase I enable highly accurate predictions. Predictions with individual HMs also achieve good results (Figure 3, Supplementary Figures S6 and S10, and Supplementary Table S3). In general, H3K27ac, H3K4me 2, H3K4me3 and H39Kac are more reliable predictors; however, the predictive ability of the combined or individual HMs varies across cell lines (Figures 3 and 4, and Supplementary Figure S10). This observation was further validated by cross-cell line tests in the following section. In spite that GC content alone does not achieve high prediction accuracy, addition of GC content to other features improves their prediction power.

We examined the predictive ability of chromatin features for SP1-TF co-occupancy in four cell types (ChIP-Seq data of SP1 were not available in the A549 cell), and the co-localizations of another six TFs, including ATF3, GABP, NRSF, POL2, USF1 and YY1. The latter test involved 15 binding pairs, as shown in Supplementary Table S8. Enrichment analyses of GC content, DNase I and 11 HMs showed distinct patterns between binding events, illustrated by the given examples of SP1-BCL11A and SP1-TAF1 (Supplementary Figure S11).

We consequently constructed and tested the computational models for each TF–TF pair in each cell type. High prediction accuracies were generally obtained. For instance, all chromatin features as a whole were able to accurately predict the GABP-USF1 co-occupancy with AUC values >0.86 in the GM12878 and K562 cell lines (Supplementary Figure S12A). For the prediction of SP1-TF combinational binding events, the average accuracies were ∼0.80, 0.77, 0.80 and 0.80 in the GM12878, H1-hESC, HepG-2 and K562 cells, respectively (Supplementary Figures S13A, B and Supplementary Table S5). For the 15 TF–TF co-occupancy with ATF3, GABP, NRSF, POL2, USF1 and YY1, chromatin features enable highly accurate predictions with AUC values >0.8 in all of five cell types (Supplementary Figures S14A, B and Supplementary Table S8).

The prediction abilities with a subset of or individual chromatin features for above TF–TF co-occupancy were similar to those for CTCF-TF co-occupancy. In general, DNase I and the combination of 11 HMs are able to achieve high prediction accuracy and addition of GC content enhances their performance (Supplementary Figures S13A, B and S14A, B). H3K27ac, H3K4me 2, H3K4me3 and H39Kac perform better than other HMs (Supplementary Figures S13C, D and S14C, D). This is consistent with our previous findings about the contribution of single HM to binding affinity of individual TFs (14). In summary, all of our observations demonstrate the strong relationship between chromatin features and TF–TF co-occupancy, and the former is sufficient to model the latter genome-widely.

### Chromatin features enable predictions of TF–TF co-occupancy across different cell lines

Both chromatin modifications and TF binding profiles exhibit dynamic and cell-specific patterns. Given that chromatin features, together with GC content, have the ability in predicting TF–TF co-occupancy, we tested to what extend the chromatin-feature models could be generalized from one cell line to others.

**Figure 5.**
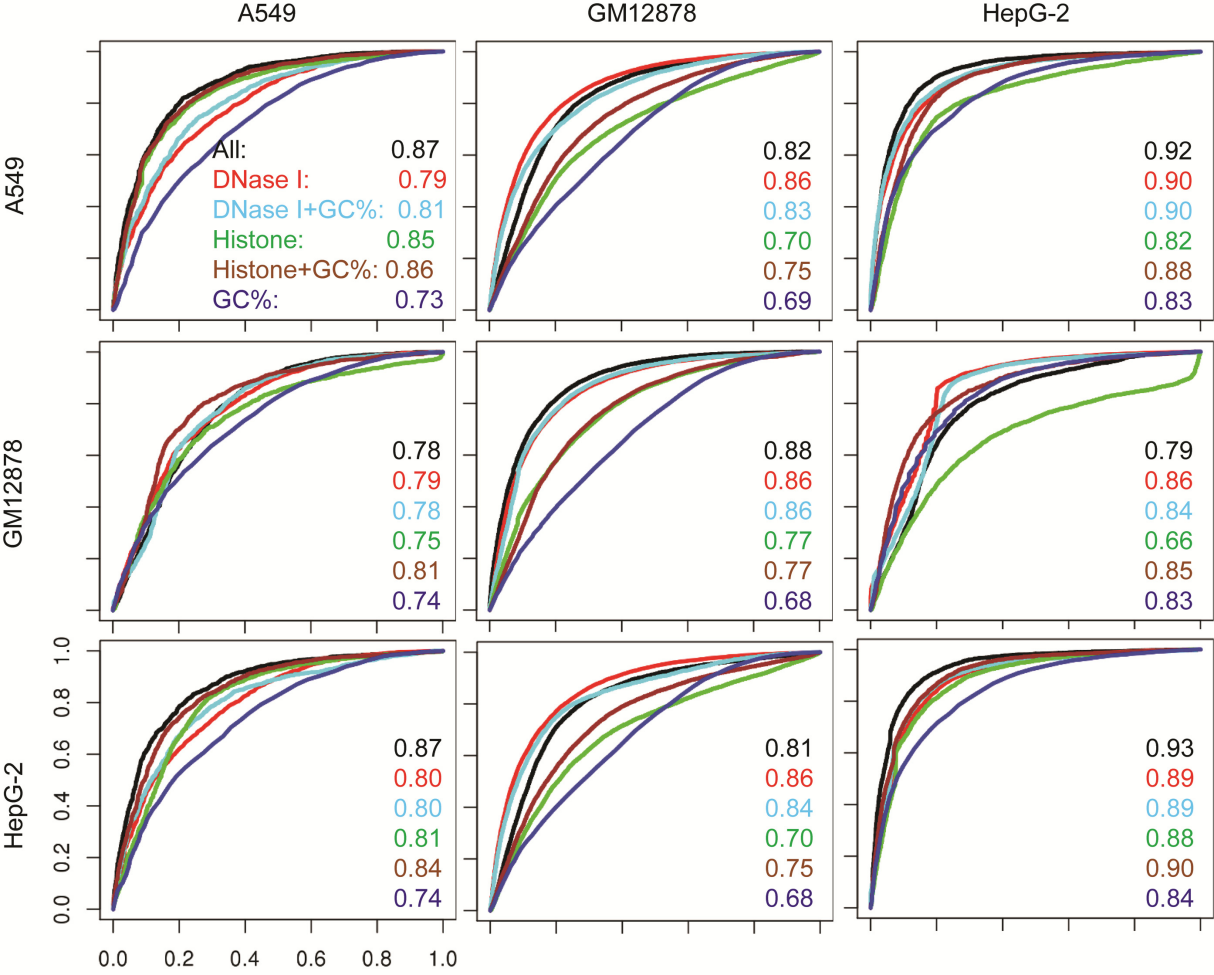
Chromatin features enable predictions of CTCF-TF co-occupancy across cell lines. Shown are ROC curves with colors representing the predictions using different chromatin features. Models were trained in the cell line indicated by row and tested on each of the five cell lines indicated by column. The AUC values are indicated on the plot as legend (see complete figures in Supplementary Figure S15).

We have constructed prediction models for all CTCF-TF pairs with 11 HMs, DNase I and GC content in the five cell lines. As shown in the Figure 3, and Supplementary Figures S6 and S10, or diagonal figures in the Figure 5 and Supplementary Figure S15, these models are able to accurately identify genome-wide binding events in the cell types they were trained on.

The models were trained on each cell line and then applied to the other four cell lines to test their prediction abilities. The results indicated that the cross-cell line applications of classifiers do not reduce their performances in predictions. For example, when we applied the models trained in the GM12878, H1-hESC, HepG-2 and K562 cell lines to the A549 cell for CTCF–YY1 predictions, the average prediction accuracies were 0.86, 0.77, 0.88 and 0.89, respectively, compared to 0.91 using the model trained by the A549 cell itself (Figure 5A and Supplementary Figure S15). The largest changes were observed with accuracies as 0.78, 0.76, 0.78 and 0.81 when models were trained within other four cells and applied to the H1-hESC cell, compared to 0.84 using model trained by the H1-hESC cell line, and *vice versa*, with accuracies as 0.77, 0.76, 0.81 and 0.79 when model was trained in the H1-hESC cell line and applied to the A549, GM12878, HepG-2 and K562 cells, compared to 0.91, 0.91, 0.91 and 0.90 when models were trained and applied to the same cell line (Supplementary Figure S15).

We did the same analyses to other CTCF-TF, SP1-TF and ATF3-/GABP-/NRSF-/USF1-TF co-occupancy. Due to the fact that not all TF binding profiles have been generated by the ENCODE project, we included various numbers of TFs in each type of cells (Supplementary Table S1). Consistent with our results from CTCF–YY1 studies, crossing-cell line applications achieved satisfactory accuracies (Supplementary Figures S12B, S16, S17 and S18). The biggest changes were seen when models from other four cell lines were applied to H1-hESC and *vice versa*. Overall, the results from our cross-cell type analyses support the generalizing associations of TF–TF co-localizations with chromatin features.

### The relationships of TF–TF co-occupancy with DNase I are more conserved

We observed that the same features had different prediction powers across cell lines (e.g. Figures 3 and 4 for CTCF–YY1), especially when comparing the H1-hESC cell with others. This was further illustrated in cross-cell line predictions (e.g. Figure 5 and Supplementary Figure S16 for CTCF–YY1). Next, we examined the conservative relationships between TF–TF co-occupancy and individual chromatin features.

**Figure 6.**
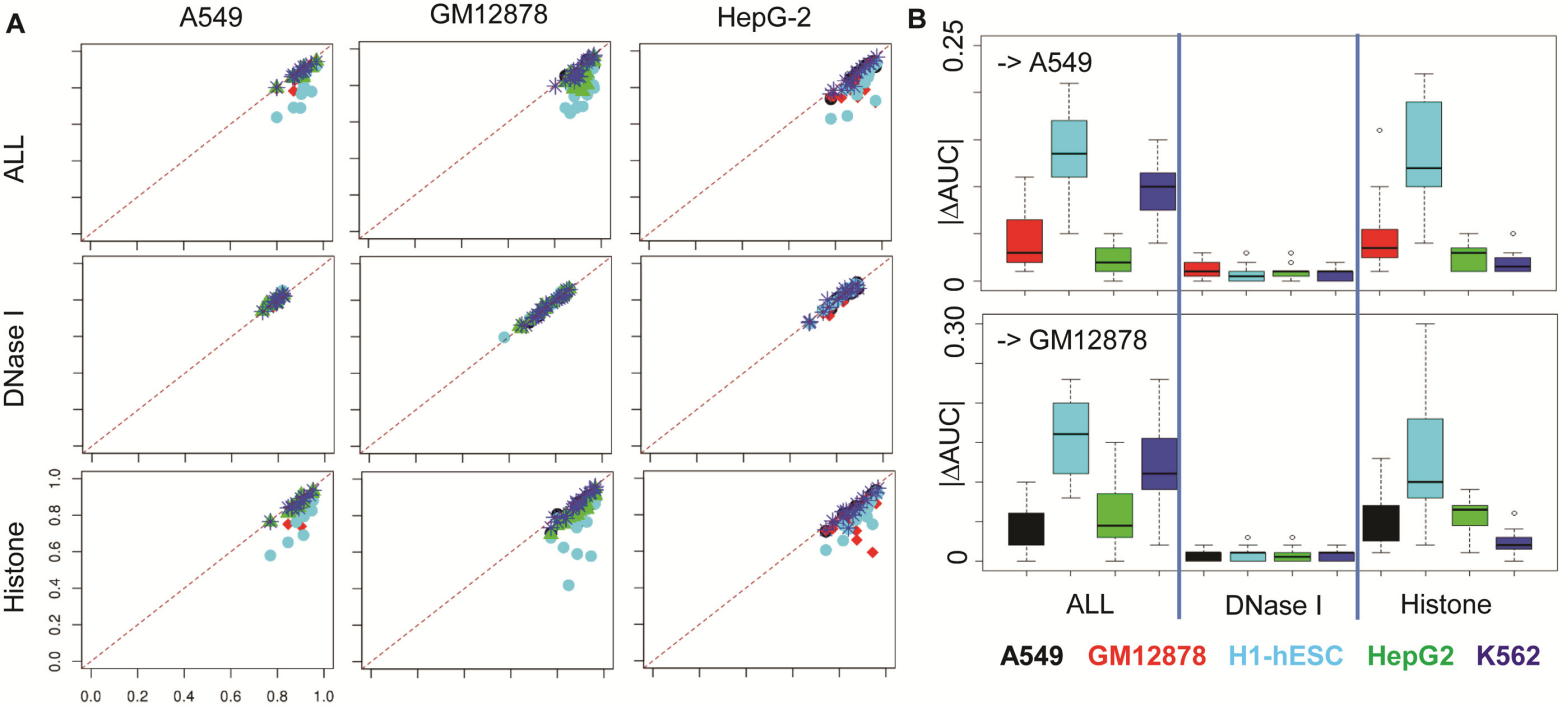
Cross cell predictions of CTCF-TF co-occupancy. (**A**) Comparisons of cross cell predictions (y-axis) to predictions using models obtained and trained in the same cell (x-axis). Test cell lines are shown by column and training cell line are indicated by colors: black, A549; red, GM12878; cyan, H1-hESC; green, HepG-2; and blue, K562. Different chromatin features were used indicated by rows. (see complete figures in Supplementary Figure S19). (**B**) Boxplots of prediction differences indicated by |ΔAUC|, in the A549 and GM12878 cell lines. |ΔAUC| was calculated by subtracting cross cell predictions using models from other cell lines, indicated by colors, from predictions using models trained from the same cell line. Different features were used in each test (see complete figures in Supplementary Figure S20).

We constructed chromatin-feature models for CTCF-TF predictions using individual or a set of chromatin features, and then applied the models obtained from one cell type to others. We found that models with DNase I, GC content and DNase I plus GC content give more consistent predictions across all of human cells (Figure 6A and Supplementary Figure S19). The overall accuracy changes (|ΔAUC|) were ∼0.02 among five cell lines, when models were built with DNase I (Figure 6B and Supplementary Figure S20). In contrast, prediction accuracies with individual or combination of 11 HMs varied across cell lines with accuracy changes ∼0.15, especially when prediction models were exchanged between H1-hESC and one of other four cell lines (Figure 6B and Supplementary Figure S20).

The above analyses were also conducted in other two sets of TF–TF pairs, and similar trends were observed (Supplementary Figures S21 and S22). Of note, for some TF–TF pairs, such as SP1-ATF2, DNase I did not enable highly accurate predictions. In summary, all results suggest that the correlations between TF–TF occupancy and DNase I/GC content are more conserved than HMs across cell lines.

## DISCUSSION

The accurate regulation of gene expression involves in a complicated interplay between TF, histone modifying enzymes and other factors. The relative importance of epigenetic modification and TF bindings in the regulation of gene expression is still under debate. Statistics analysis has revealed that these two factors regulate gene transcription in a highly coordinate manner (34), and are redundant for predicting gene expression (55). Several studies have described direct interactions between histone modifying enzymes and TFs (56). Co-occupancy of a binding site by multiple TFs plays a critical role in fine regulation of gene expression (57). Certain patterns of histone marks have been observed around co-binding sites of some TF pairs, such as FOXA1-FOXA3 (57), YY1-MYC and CTCF-NF-Y (17). However, there are no approaches available currently for computationally identifying TF–TF co-occupying sites using chromatin features and quantitatively modeling the correlation between them. We here introduced chromatin features, that are always cell-type specific, to refine the description of TF–TF co-occupancy, and observed a strong correlation of TF–TF co-occupancy with chromatin features. This relationship was further confirmed by quantitative predictions of TF–TF co-occupancy using multiple or individual chromatin features.

Experimental techniques such as ChIP-Seq have been used to identify TF–TF co-occupancy (23,32,58–60). Unfortunately, these experimental methods are always expensive and time-consuming. Meanwhile, computationally predicting models using sequence-based PWM methods (61–63) or combing ChIP-Seq data and PWMs (64) have also been developed to detect the co-occurring TFs (63) and their binding motifs (64–66). However, prediction of the putative TFBSs using the predefined PWM suffers from a high rate of false positive discovery (67). Moreover, these approaches ignore the influence from cell conditions, which are crucial for epigenetic modifications, chromatin accessibility, TF binding and consequently gene regulation (11,14,17,34,42,68). As a result, the prediction accuracy could vary greatly across cell types. For instance, the *cobindR* software (69) uses PWMs to identify the co-occurring TFs and their binding sites. This sequence-based approach can detect 6,444 CTCF–YY1 co-occupying sites (PWMs obtained from http://jaspar.genereg.net (70)), which cover 49, 20, 31, 19 and 26% sites obtained from the ChIP-Seq data (Supplementary Table S1) in the A549, GM12878, H1-hESC, HepG-2 and K562 cells, respectively. As a comparison, our method used cell type-specific chromatin features as predictors, which largely improve predictive accuracy for cell type-specific TF–TF co-occupying sites (Supplementary Table S3). Of noting, the most importance is that our study illustrated the correlation between chromatin features and TF–TF co-occupancy, which is the main aim of this study and can improve our understanding of the interactions between epigenetic and genomic regulation.

The co-occupied TFs may have different regulation functions from solely bound TF. For example, co-localizations of CTCF and YY1 can enhance transcriptional activity of genes that they are co-occupied (19). Analysis showed that the binding intensities of CTCF at regions co-occupied by YY1 are significantly greater than those bound by only CTCF (Student's *t*-test *P* < 1e-17 in the five cell lines; Supplementary Figure S23), indicating their differentially functional effects. By profiling co-occupancy of CTCF–YY1 and other TF–TF pairs with chromatin features, we demonstrated the important roles of chromatin modifications in gene regulation and the strong associations between genetic and epigenetic regulations.

Our analysis further illustrated the generalization of this correlation across cell types, which led to the possible application of a prediction model trained from one cell line using combination of chromatin features to other cells for accurate predictions of TF–TF co-occupancy. When applied individual chromatin features in our models, the associations of DNase I with TF–TF co-occupancy were very conserved, and the cross-cell type applications of models with DNase I did not result in dramatically changes of prediction accuracy. This observation suggested that, although DNase I shows distinct profiling patterns in different cell types (68), these patterns may coordinately change with TF–TF interactions regardless of cell conditions. In contrast, the associations of individual or the combined 11 HMs are less conserved among cell lines. This may be explained by the reported cell-specific correlations between HMs and individual TF binding affinities (14).

TF–TF co-occupancy may have an effect on transcriptional output. Comparisons of the expression levels for RefSeq genes showed that the genes overlapped YY1-only binding events are significantly more highly expressed, followed by genes overlapped with CTCF–YY1 binding events, in contrast to genes overlapped CTCF-only binding events (Wilcoxon rank-sum test, *P*-values < 10e-8; Supplementary Figure S24A), consistent with the previous findings (19). This observation indicates the different functions of CTCF–YY1 co-occupying regions compared to others.

We further tested whether transcriptional output has relationship with both TF–TF co-occupancy and chromatin features, such as DNase I. Comparisons showed that, although either CTCF–YY1 co-occupancy or DNase I is associated with transcriptional activity (17,19,68), the combination of CTCF–YY1 and DNase I did not necessarily lead to higher transcriptional outputs (Supplementary Figure S24B). Indeed, even if CTCF–YY1 and DNase I occurred in the same genomic regions, such as gene promoters, the transcriptional output varied from gene to gene (Supplementay Figures S24C, D). This may be explained by the complicated correlation between DNase I and gene expression. For instance, Wang *et al*. showed that, even for the similarly expressed genes, the distribution of DNase I may differ among different chromosomes (71).

DNA methylation is another type of epigenetic modification involved in the regulation of gene expression, cell growth and disease development (6,72). Early studies reported that DNA methylation is related with TF binding (73), but it alone is not sufficient to prevent protein binding (74–76), or had a weak correlation with individual TF binding affinity (14). We examined the relationship between DNA methylation (see ‘Materials and Methods’ section) and TF–TF co-occupancy. We selected the methylation level of CpG site(s) mapped into the 100-bp bin centered at each TFBS to compute methylation level at that binding site. Most of TFBSs do not have methylated CpG site(s). In the GM12878 cell lines, 5583 out of 40 247 CTCF binding sites have ≥1 CpGs, including 2,415 CTCF–YY1 and 3148 CTCF-only sites. We constructed SVM classifier with DNA methylation and/or other chromatin features. The results showed that DNA methylation has very fair predication ability, with accuracies ∼0.57 in the five human cell lines. Moreover, the combinations of DNA methylation with any other chromatin features led to nearly same predictive performances (Supplementary Figure S25).

TFs prefer working together to regulate gene expression by targeting the same genomic regions, namely TF hotspots (21,22,28,77). These regions are usually cell type specific, represented by active histone marks and reflect certain chromatin states (77,78). We therefore examined the correlation between chromatin states and TF–TF co-occupancy. Since the CTCF ChIP-Seq has been used by the ChromHMM for the determination of chromatin state segmentation, we selected the SP1-TF pairs as the testing examples. TFBSs were mapped into the 15 chromatin states with different distributions and the association of SP1-TF co-occupancy with all 15 chromatin states were assessed (Supplementary Figure S26). In general, the models using the chromatin state segmentation gave predictive outcomes with accuracies lower than the ones using the combination of all chromatin features or the 11 HMs (Supplementary Figures S27A, B). This suggested that TF–TF co-occupancy is not only reflected by chromatin states or those HMs used for chromatin state determination, but other chromatin features, such as DNase I and GC components. As well, the adding of chromatin state segmentation to any other chromatin features did not significantly change the predictive outcomes (Supplementary Figure S27A, C). This may be because that, during the prediction of chromatin state segmentation with the ChromHMM model, 8 of the 11 HMs from our study have been used as the inputs, and therefore the information from the chromatin state segmentation is partially overlapped with the one from the 11 HMs.

In summary, we have presented a statistical and computational approach to investigate the complicated interplays between genetic and epigenetic regulations of gene expression. Although our analysis cannot demonstrate the relative importance or causative role of TFs or chromatin features in transcriptional regulation, we have elucidated a strong relationship between TF–TF co-occupancy and various chromatin features through a large-scale statistical and computational experiments, which will help us in understanding the mechanisms of combinational regulatory landscape.

## SUPPLEMENTARY DATA

Supplementary Data are available at NAR Online.

SUPPLEMENTARY DATA
