# Supplementary material for: Modeling co-occupancy of transcription factors using chromatin features
Source: Nucleic Acids Res. 2015 Nov 20;44(5):e49. doi: 10.1093/nar/gkv1281 (PMC4797273; doi:10.1093/nar/gkv1281)
Supplement: SUPPLEMENTARY DATA [file supp_44_5_e49__index.html]

Modeling co-occupancy of transcription factors using chromatin features — Modeling co-occupancy of transcription factors using chromatin features — SUPPLEMENTARY DATA 

# Modeling co-occupancy of transcription factors using chromatin features

## SUPPLEMENTARY DATA

- SUPPLEMENTARY DATA
- SUPPLEMENTARY DATA
