# Supplementary material for: Modeling co-occupancy of transcription factors using chromatin features
Source: Nucleic Acids Res. 2015 Nov 20;44(5):e49. doi: 10.1093/nar/gkv1281 (PMC4797273; doi:10.1093/nar/gkv1281)
Supplement: SUPPLEMENTARY DATA [file supp_gkv1281_nar-02445-met-n-2015-File008.docx]

**Modeling Co-occupancy of Transcription Factors Using Chromatin Features**

Liang Liu^1^, Weiling Zhao^1^, Xiaobo Zhou^1,*^

^1^ Center for Bioinformatics and Systems Biology and Department of Radiology, Wake Forest School of Medicine, Winston-Salem, NC 27157, USA

* To whom correspondence should be addressed. Tel: +1-336-713-1789; Fax: +1-336-713-5891; Email: [xizhou@wakehealth.edu](mailto:xizhou@wakehealth.edu)

**Supporting Information**

1. **Supplemental Materials**
2. **Supplemental Tables S1-S8 (See Supplemental Excel Files)**
3. **Supplemental Figures S1-S27**

**1. SUPPLEMENTAL MATERIALS**

**Number of binding sites.** Various numbers of binding sties exist for CTCF across different cell lines, ranging between 38,133 (in A549) and 55,778 (in HepG-2) (Supplemental Table S1). Following the same procedure ([1](#_ENREF_1)), binding sites for other types of TFs were also identified, the numbers of which vary from 1,116 (FOXL1 in H1-hESC) to 46,367 (MAX in K562). These TFs may bind at the same regions with CTCF (Additional file 3: Figure S1A). Distinct preferences of co-occupancy with CTCF were established. For instance, YY1 prefers to co-exist with CTCF, and ~30% of its binding sites were co-occupied by CTCF (Additional file 3: Figure S1A and Supplemental Table S1); in contrast, PU1 only shares only ~7% binding regions with CTCF (Supplemental Table S1).

**Sequence and chromatin features of different binding events.** We examined the sequence features among binding sites of CTCF and YY1 as an example. For the binding events involved CTCF, including CTCF-only (CTCF binding regions without detected YY1 binding sites), CTCF-YY1 (CTCF and YY1 co-occupying genome regions) and YY1-only (YY1 binding regions without detected CTCF binding sites) binding regions, we analyzed DNA sequences surrounding binding sites of CTCF and YY1, determined by summits of ChIP-Seq peaks. We used the top 1000 binding sites in each type of binding events (CTCF-only, CTCF-YY1 and YY1-only) to identify the canonical motifs and discovered the *de novo* motifs using MEME tool ([2](#_ENREF_2)). We observed that the binding motifs for CTCF or YY1 in the three binding categories kept similar (Additional file 3: Figure S3). This observation keeps consistence with previous findings ([3](#_ENREF_3)), indicating that DNA sequence may not be the determinant for the co-localizations of CTCF and YY1.

GC content dictated nucleosome depletion at mammalian promoters ([4](#_ENREF_4),[5](#_ENREF_5)), and GC-richness appeared to be beneficial for TF binding ([5](#_ENREF_5)). We therefore analyzed the role of GC content in differentiating binding events. We selected the fragments located in the 100-bp region centered at ChIP-Seq peak summits, and then calculated GC content in each DNA fragment. The results showed that CTCF-YY1 co-occupying regions are significantly more associated with GC content than CTCF-only binding sites (Student’s *t* test *p* < 1e-15; Additional file 3: Figure S4), suggesting the co-occupied regions may have a stronger transcriptional activities ([3](#_ENREF_3),[4](#_ENREF_4),[6](#_ENREF_6)).

We also investigated chromatin features at binding sites. The HM patterns at TFBSs were characterized by 11 types of histone methylation and acetylation, each of which has been associated with transcriptional activation, suppression, or both ([7-9](#_ENREF_7)). Significant differences were found between CTCF-YY1 co-occupying and CTCF-/YY1-only binding regions (Additional file 3: Figure S5). All HMs, except H3K27me3, were highly enriched in CTCF-YY1 co-occupying regions than these in the CTCF-only binding sites. This is consistent with the association between co-localizations of the two TFs and transcription activity; on the contrary, H3K27me3 is associated with the suppression of transcription in a cell type-specific manner ([10](#_ENREF_10),[11](#_ENREF_11)), as well as other HMs, such as H3K4me1 ([12](#_ENREF_12)), H3K4me3 ([13](#_ENREF_13)), and H3K27ac ([14](#_ENREF_14)).

We specifically analyzed HM signals in a smaller 100-bp region. We found that enrichment of chromatin features were significantly different among binding events (Student’s *t* test *p* < 1e-15; Additional file 3: Figure S4). The tag densities of H2A.z, H3K27ac, H3K9ac, H3K4me2, H3K79me2, H3K4me1 and H3K4me3 were significantly higher in the CTCF-YY1 co-occupying regions than those found in the CTCF-only binding sites; other features likely to have the opposite outcomes.

DNase I, a chromatin feature indicating accessibility of DNA sequence/chromatin zones and functional related to transcriptional activity ([15](#_ENREF_15),[16](#_ENREF_16)), was found to have high predictive power for TFBSs ([17](#_ENREF_17),[18](#_ENREF_18)). We analyzed the DNase I accessibility profile for different types of binding events. Supplemental Figure S5 show that the CTCF-YY1 co-occupying regions generally have higher DNase I signals than those in the CTCF-only binding regions, indicating chromatin is more accessible. This trend was even stronger when considering the smaller 100-bp region centered at peak summits (Additional file 3: Figure S4). Across the 9 cell lines, higher DNase-Seq signals were presented at the region shared by CTCF and YY1 (Student’s *t* test *p* < 1.0e-15).

We examined the all CTCF-TF composition in the 5 cell lines, and obtained similar results (student’s *t* test *p* < 0.05; Supplemental Table S2). It is worth to note that minor differences exist in chromatin feature profiles between CTCF-TF and TF-CTCF binding events, because the binding sites of co-occupied CTCF and its partner, representing by ChIP-Seq peak summits, are not located at the exactly same genome positions. Taken together, these results suggested that chromatin features have strong correlations with CTCF-TF binding events, which led us to construct computational chromatin feature-model that enables to discriminate CTCF-TF co-occupying genome binding regions from CTCF-only or TF-only binding sites.

**2. Supplemental Tables (See Supplemental Excel Files)**

**Supplemental Table S1:** Number of TF-TF co-occupying and TF-only binding events in the five human cell lines. CTCF-TF: genome regions co-occupied by CTCF and another TF, such as YY1; CTCF-only: genome sites bound by only CTCF; TF-only: genome regions bound only by another TF.

**Supplemental Table S2:** Comparisons of chromatin signals between CTCF-TF co-occupying and CTCF-/TF-only binding events. Student's t-test was performed for each comparison, and a -log10 transformation was applied to *p* value. Comparisons with *p* value > 0.05 (-log10(0.05) < 1.30) were highlighted.

**Supplemental Table S3:** Prediction accuracies of CTCF-TF co-occupancy in the (A) A549, (B) GM12878, (C) H1-hESC, (D) HepG-2, and (E) K562 cell lines.

**Supplemental Table S4:** Cross-cell line tests by applying models obtained from one of the five cell types to the (A) A549, (B) GM12878, (C) H1-hESC, (D) HepG-2, and (E) K562 cell lines, for the predictions of CTCF-TF co-occurrence.

**Supplemental Table S5:** Prediction accuracies of SP1-TF co-occupancy in the (A) GM12878, (B) H1-hESC, (C) HepG-2, and (D) K562 cell lines. The A549 cell line does not have ChIP-Seq data for SP1 available.

**Supplemental Table S6:** Cross-cell line tests by applying models obtained from one of the five cell types to the (A) GM12878, (B) H1-hESC, (C) HepG-2, and (D) K562 cell lines, for the predictions of SP1-TF co-occurrence.

**Supplemental Table S7:** Prediction accuracies of ATF3-/GABP-/NRSF-/POL2-/USF1-TF co-occupancy in the (A) A549, (B) GM12878, (C) H1-hESC, (D) HepG-2, and (E) K562 cell lines.

**Supplemental Table S8:** Cross-cell line tests by applying models obtained from one of the five cell types to the (A) GM12878, (B) H1-hESC, (C) HepG-2, and (D) K562 cell lines, for the predictions of ATF3-/GABP-/NRSF-/POL2-/USF1-TF co-occurrence.

**2. Supplemental Figures**

**
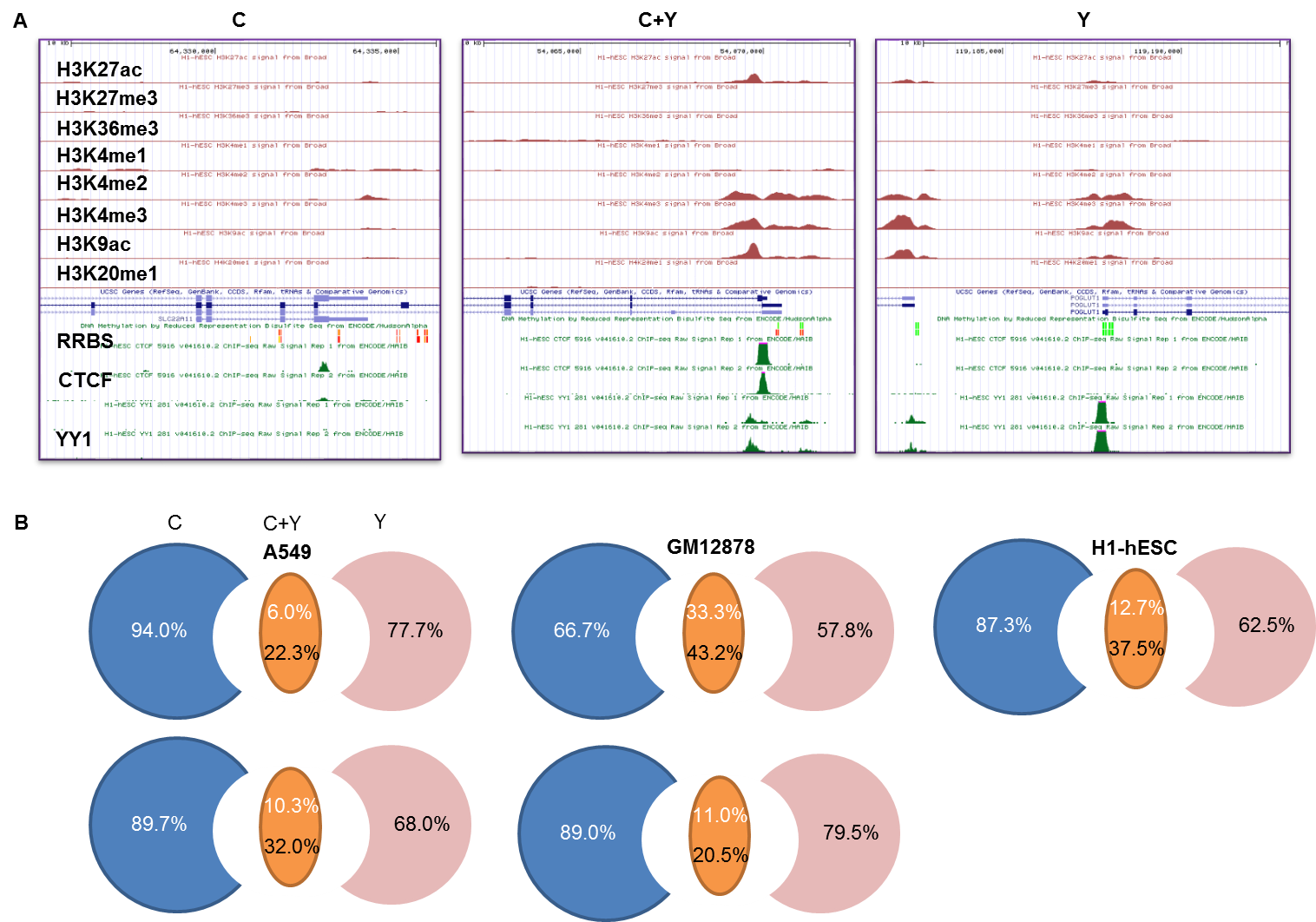
**

**Supplemental Figure S1:** Co-occupied and solely binding events of CTCF and YY1. (A) CTCF and YY1 binding events, including CTCF-only binding (C), CTCF-YY1 co-occupying (C+Y), and YY1-only binding (Y) events. (B) The percentages of three binding events (C, C+Y and Y) in the five cell types (Table S1). Binding sites were obtained from ChIP-Seq data and generated with the UCSC genome browser.

**
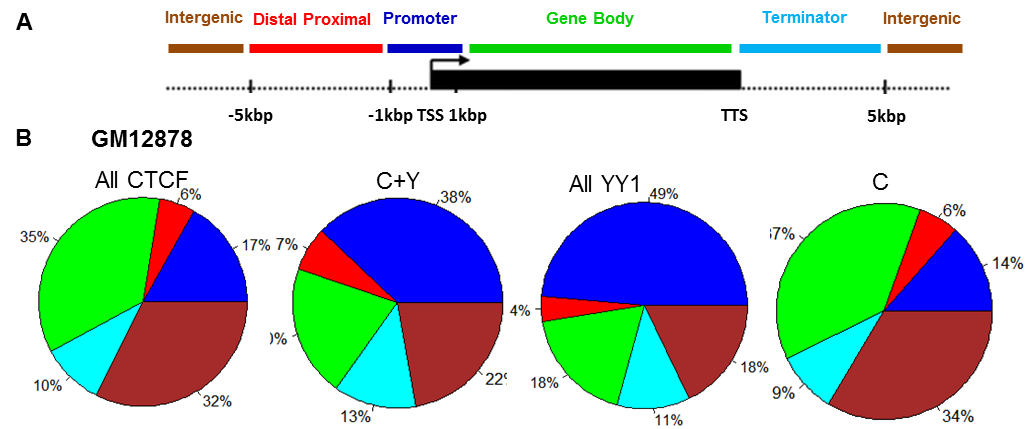
**

**Supplemental Figure S2:** (A) Functional annotation of DNA sequence based on UCSC RefGene coordinates. (B) Distributions of CTCF/YY1 binding sites across different binding events in different genomic regions.

**
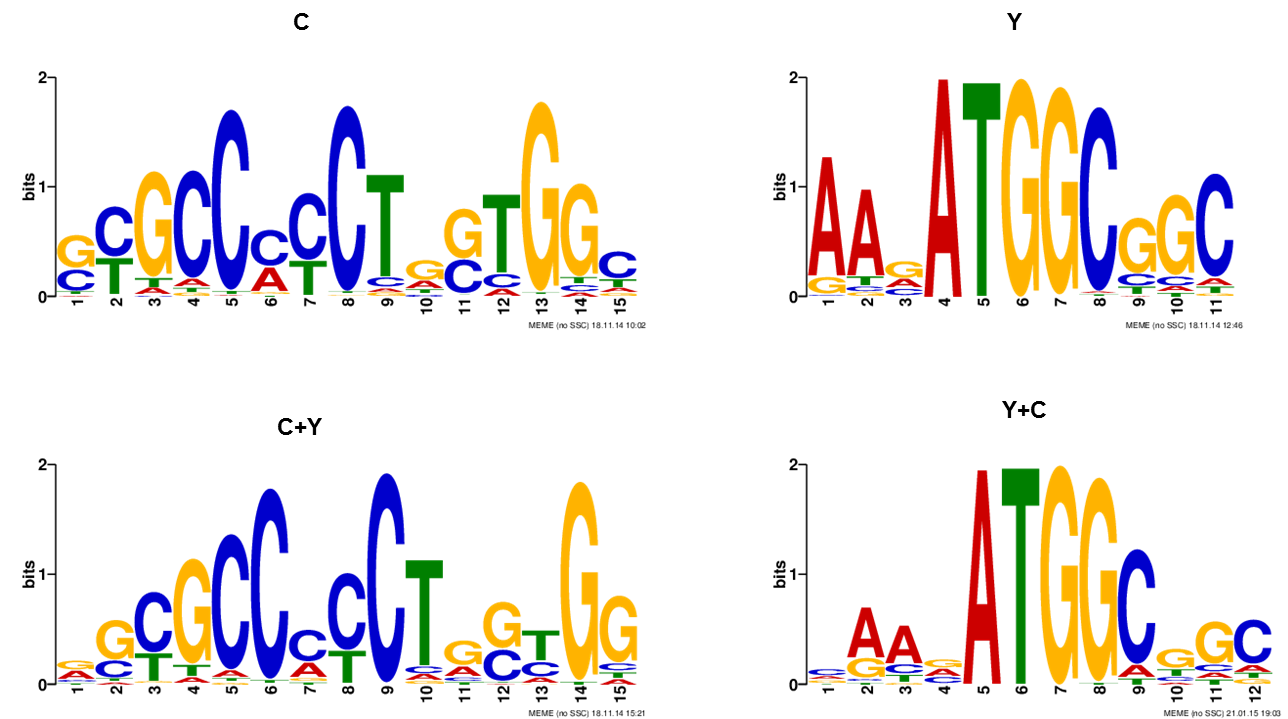
**

**Supplemental Figure S3:** Motifs of CTCF identified at CTCF-only (C) and CTCF-YY1 co-occupying (C+Y) sites, and of YY1 identified at YY1-only (Y) and YY1-CTCF co-occupying (Y+C) sites in the A549 cell line.

**
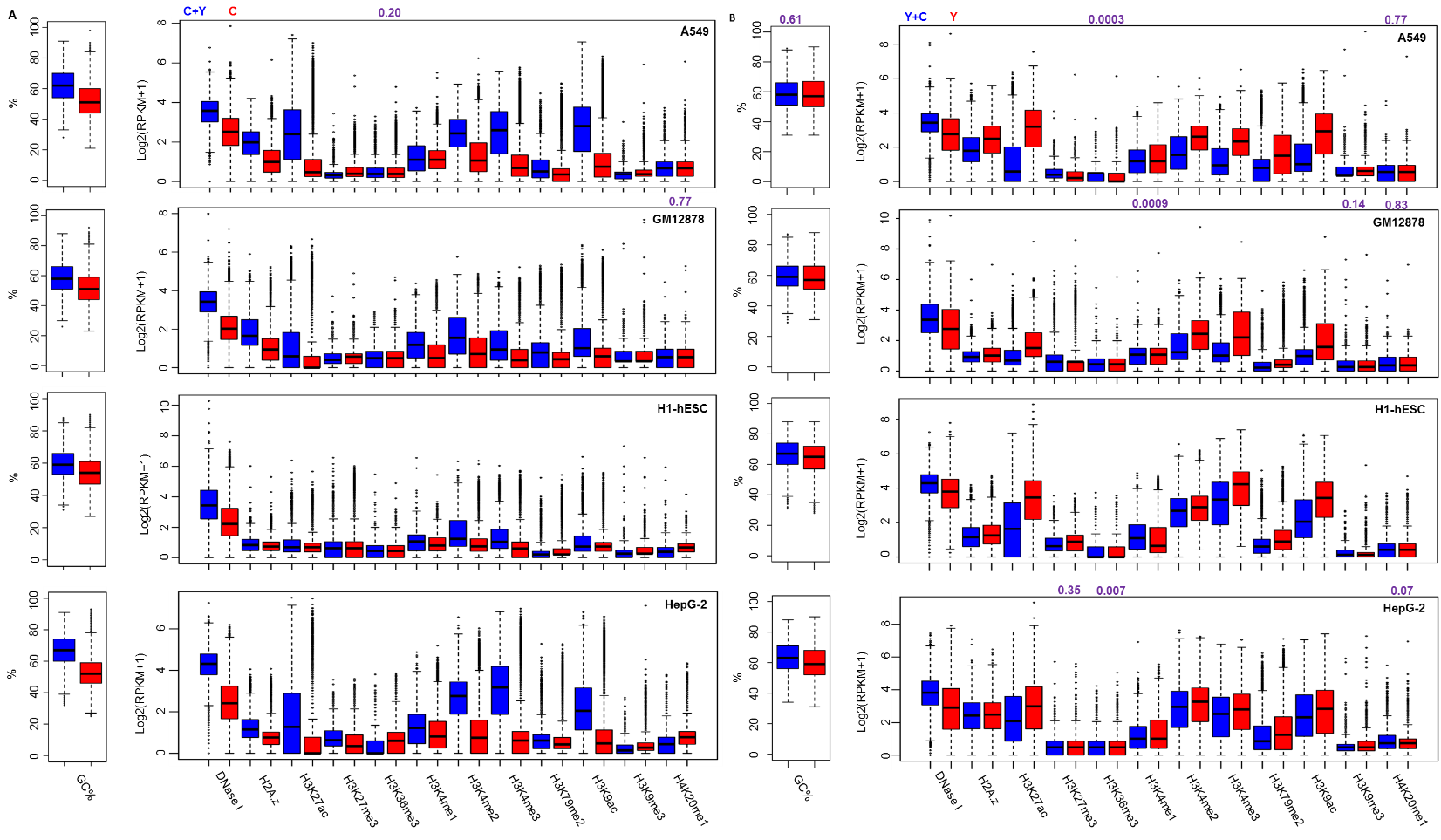
**

**Supplemental Figure S4:** Comparison of chromatin feature profiles (A) between CTCF-YY1 co-occupying (C+Y, blue) and CTCF-only binding regions (C, red), and (B) between YY1-CTCF (Y+C, blue) and YY1-only (Y, red) binding regions, in the A549, GM12878, H1-hESC and HepG-2 cell lines. Student’s *t* test was performed to each comparison. All tests reached *p* values < 1.0E-5, unless values are shown in figures (numbers in purple).

**
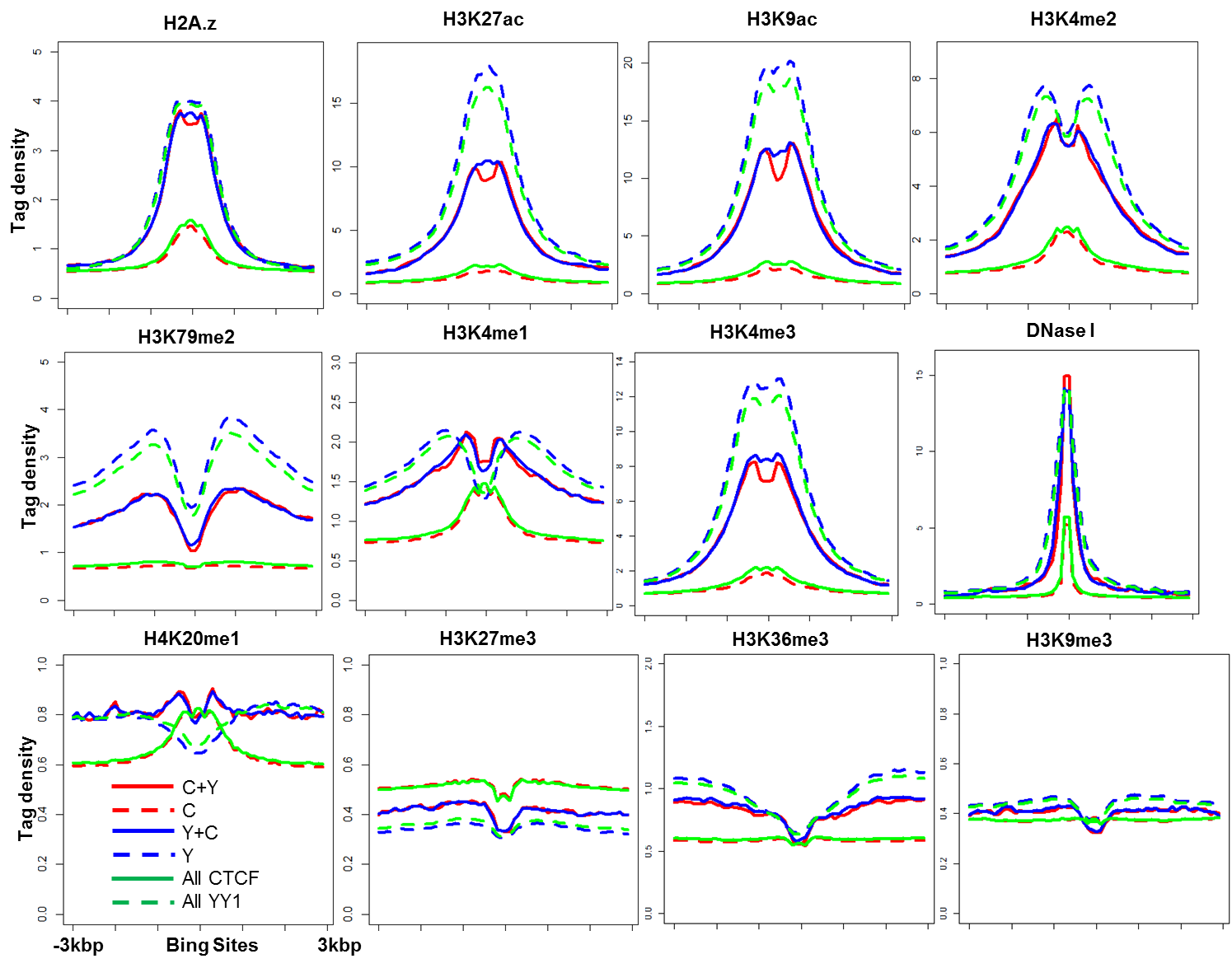
**

**Supplemental Figure S5:** Chromatin profile patterns in the upstream and downstream 3k-bp regions surrounding CTCF-YY1 (C+Y), CTCF-only, YY1-CTCF (Y+C), YY1-only (Y), all CTCF and YY1 binding events, with each represented by different types of lines (shown as legend). Slight differences exist between profiles of C+Y and Y+C because of the distances between the binding sites of CTCF and YY1, although they co-occupy the same genome regions.

**
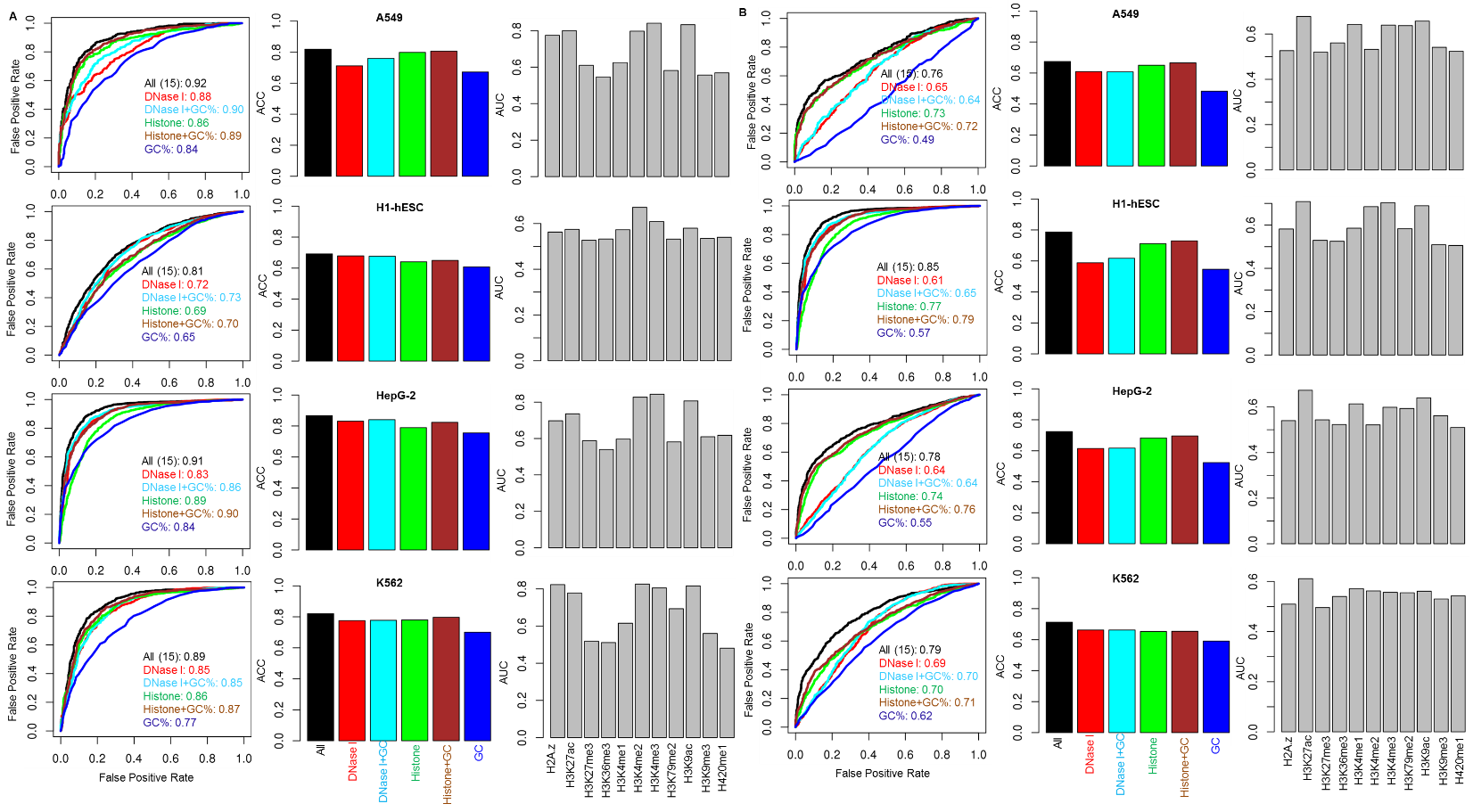
**

**Supplemental Figure S6:** Chromatin features are predictive of CTCF-YY1 co-occupancy from (A) CTCF-only and (B) YY1-only events with high accuracies in the A549, H1-hESC, HepG-2, and K562 cell lines. Left: ROC curves are shown with colors representing predictions using different chromatin features, and AUC values are indicated in the legend; Middle: Predictions evaluated with ACCs; and Right: Predictions evaluated with AUC values using individual histone features.

**
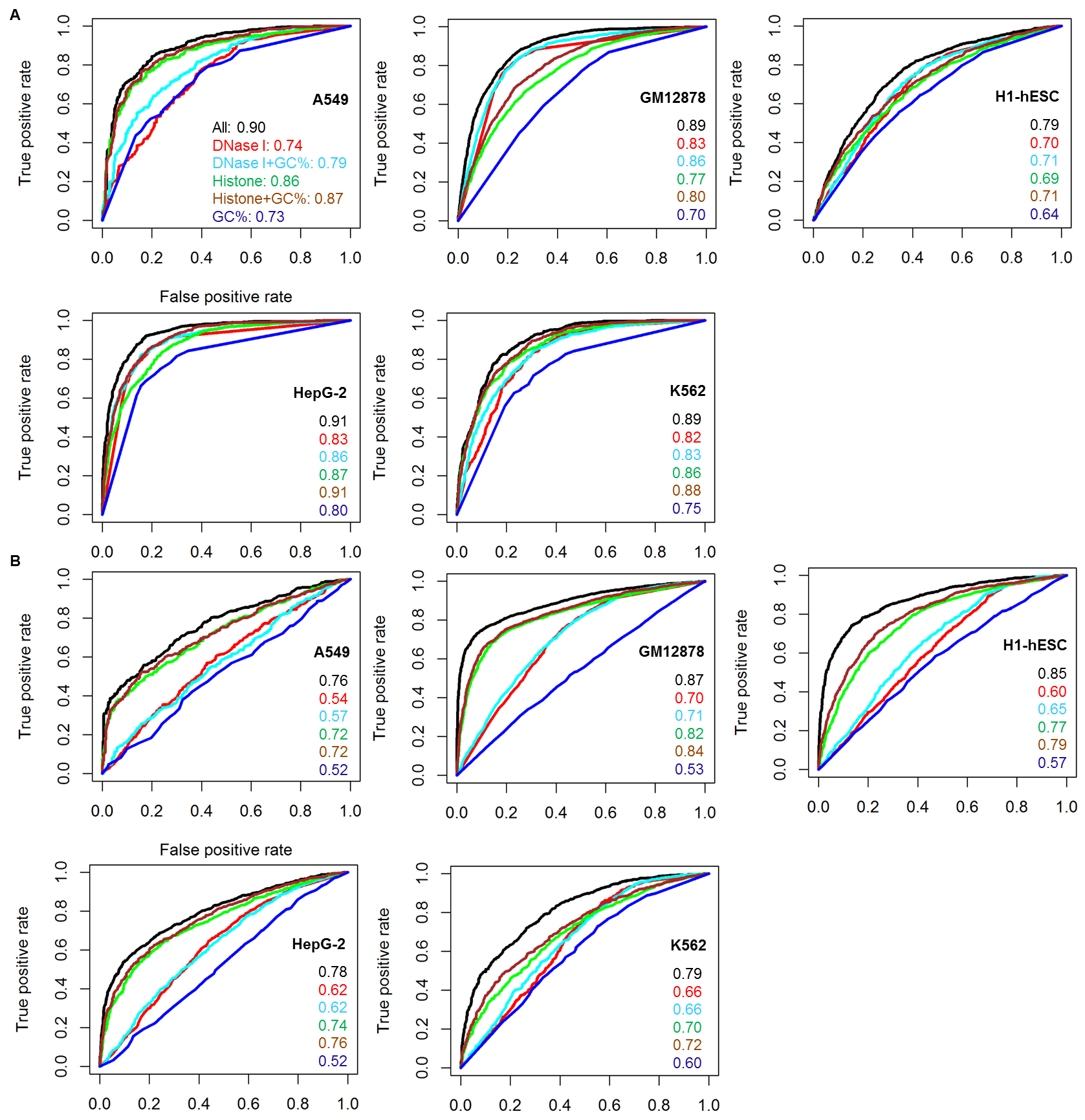
**

**Supplemental Figure S7:** Chromatin features are predictive of CTCF-YY1 co-occupancy from (A) CTCF-only and (B) YY1-only events with high accuracies in the A549, GM12878, H1-hESC, HepG-2, and K562 cell lines. ROC curves are shown with colors representing predictions using different chromatin features, and AUC values are indicated in the legend. Random Forest Classifier was employed.


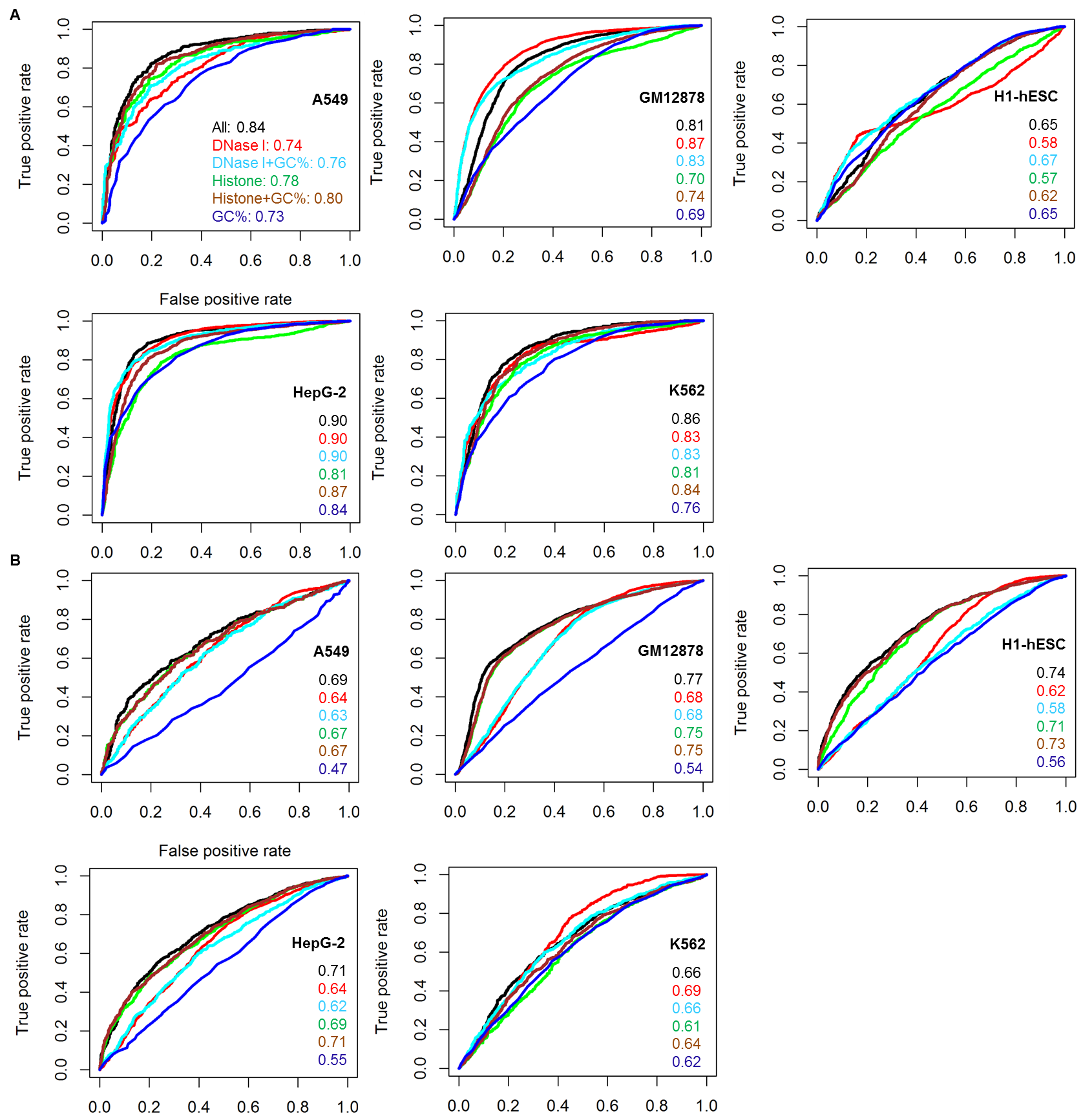


**Supplemental Figure S8:** Chromatin features are predictive of CTCF-YY1 co-occupancy from (A) CTCF-only and (B) YY1-only events with high accuracies in the A549, GM12878, H1-hESC, HepG-2, and K562 cell lines. ROC curves are shown with colors representing predictions using different chromatin features, and AUC values are indicated in the legend. Naïve Bayes Classifier was employed.


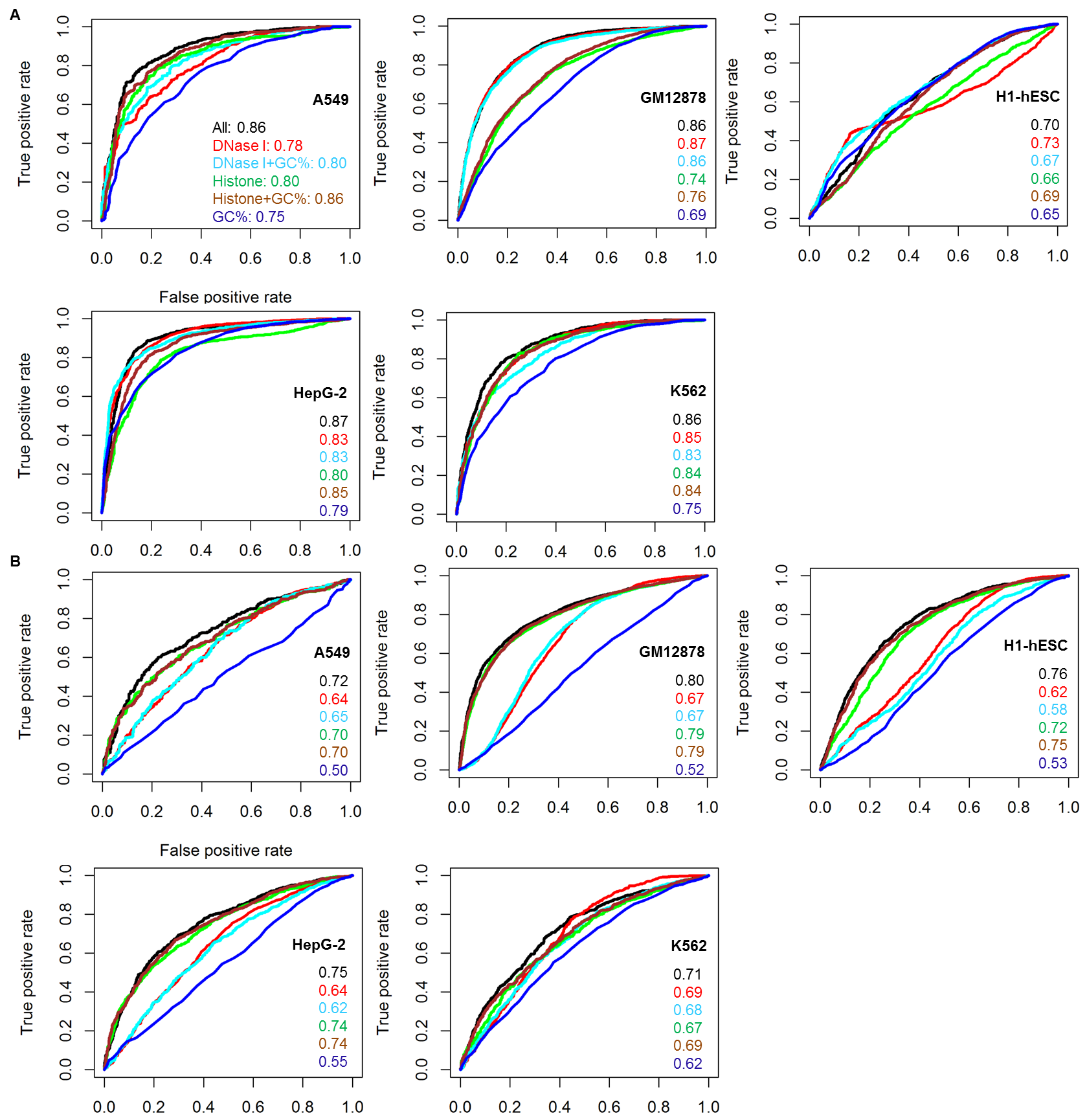


**Supplemental Figure S9:** Chromatin features are predictive of CTCF-YY1 co-occupancy from (A) CTCF-only and (B) YY1-only events with high accuracies in the A549, GM12878, H1-hESC, HepG-2, and K562 cell lines. ROC curves are shown with colors representing predictions using different chromatin features, and AUC values are indicated in the legend. Linear Discriminant Analysis was employed.


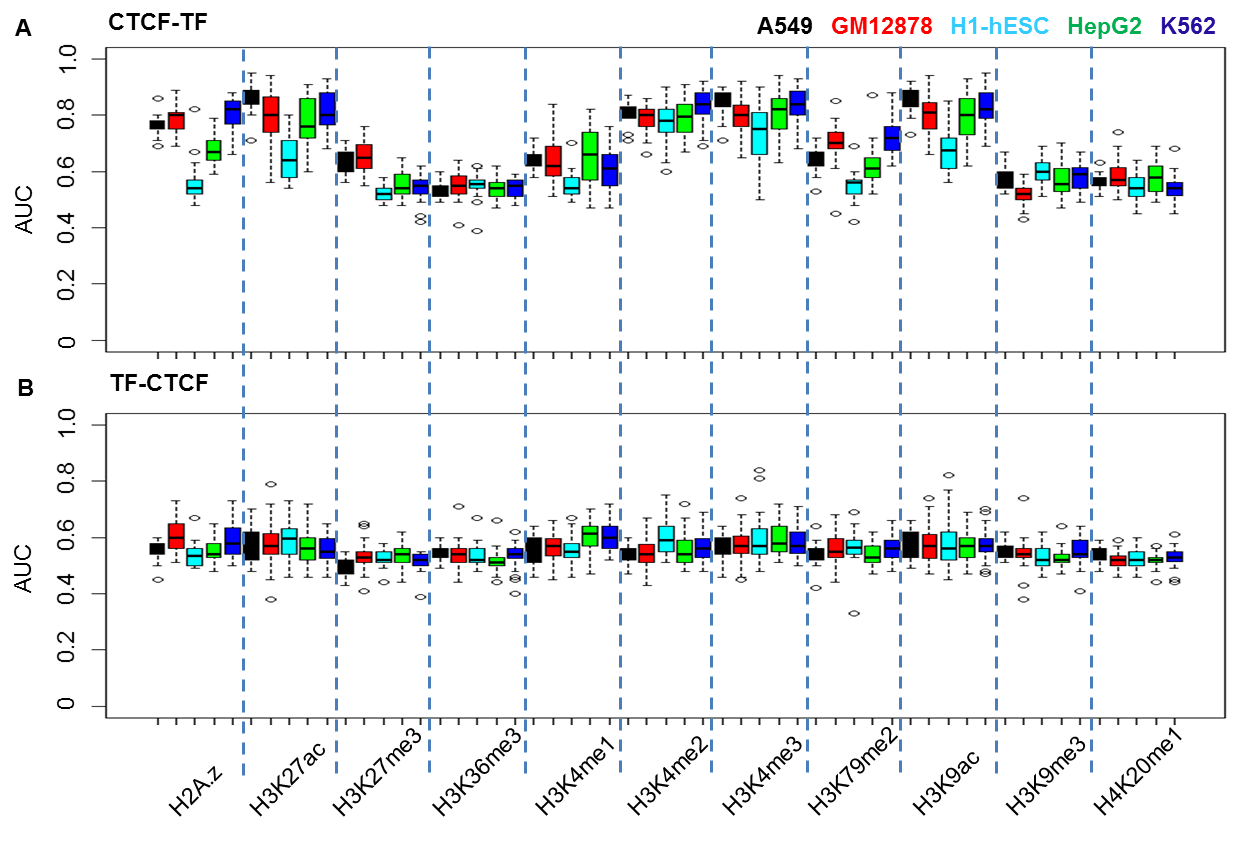


**Supplemental Figure S10:** Chromatin features are predictive of CTCF-YY1 co-occupancy from (A) CTCF-only and (B) YY1-only events with high accuracies in the 5 human cell lines. Computational models were trained and applied to the same CTCF-TF pair in the same cell line, indicated by colors as legend: black, A549; red, GM12878; cyan, H1-hESC; green, HepG-2; and blue, K562. Models were trained using individual HMs. Various number of TFs were included for each cell type due to the availability of ChIP-Seq data.

**
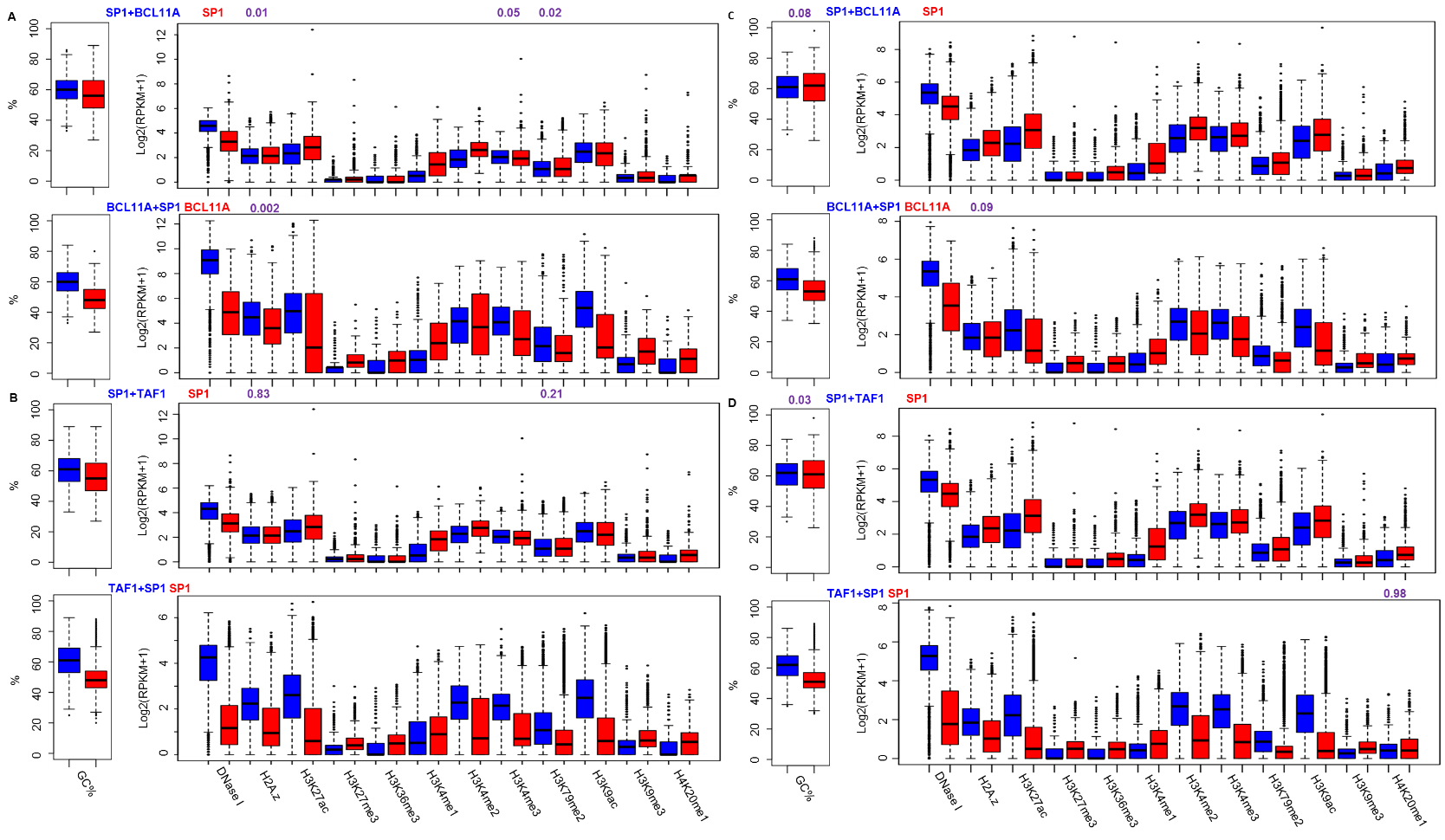
**

**Supplemental Figure S11:** Comparison of chromatin feature profiles (A, C) between SP1-BCL11A co-occupying (blue) and SP1-/BCL11A-only binding (red) regions, and (B, D) between SP1-TAF1 co-occupying (blue) and SP1-/TAF1-only (red) events in the (A, B) GM12878, and (C, D) K562 cell lines. Student’s *t* test was performed to each comparison. All tests reached *p* values < 1.0E-5, unless values are shown in figures (numbers in purple).

**
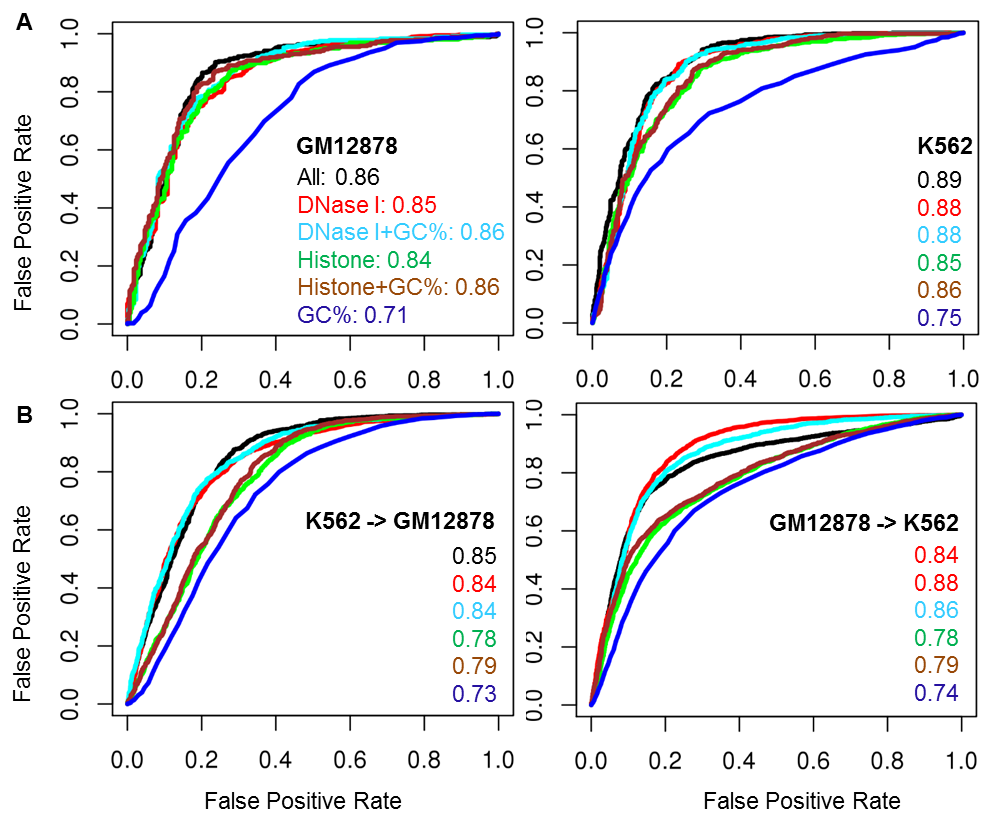
**

**Supplemental Figure S12:** Chromatin features are predictive of GABP-USF1 co-occurrence. (A) The computational models were trained and applied in the same GM12878 or K562 cell lines, and (B) the models were trained in the K562 or GM12878 cell lines and applied to the GM12878 or K562 cell lines, respectively. ROC curves are shown with colors representing predictions with different chromatin features.

**
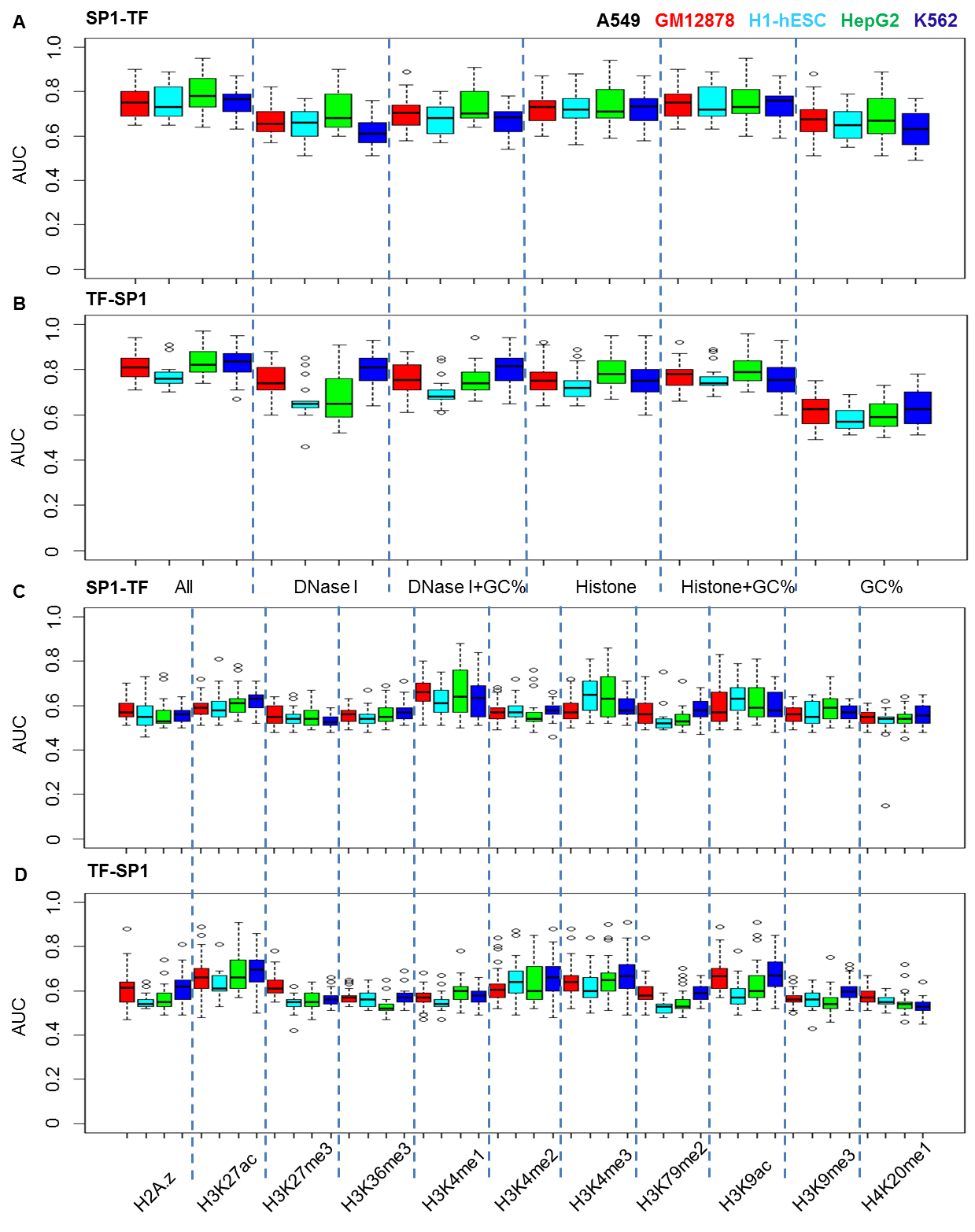
**

**Supplemental Figure S13:** Chromatin features are predictive of SP1-TF co-occurrence. Chromatin features predict SP1-TF co-occupancy from (A, C) SP1-only and (B, D) TF-only events with high accuracies. Computational models were trained and applied to the same SP1-TF pair in the same cell line, indicated by colors as legend: red, GM12878; cyan, H1-hESC; green, HepG-2; and blue, K562. Different chromatin features were included in each test: (A, B) the combinations of all or a few features, and (C, D) individual HMs.

**
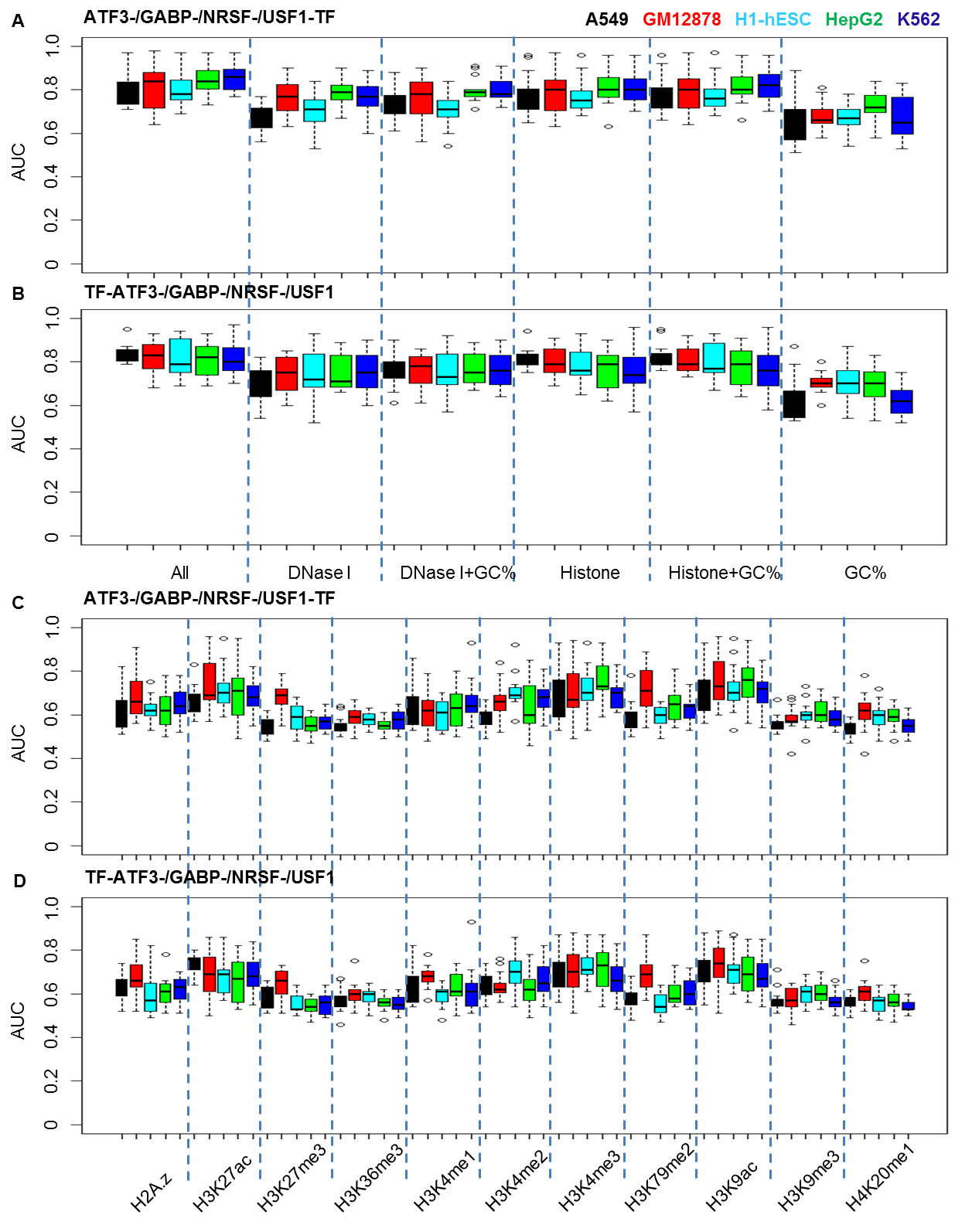
**

**Supplemental Figure S14:** Chromatin features are predictive of ATF3-/GABP-/NRSF-/USF1-TF co-occurrence. Chromatin features predict ATF3-/GABP-/NRSF-/USF1-TF co-occupancy from (A, C) ATF3-/GABP-/NRSF-/USF1-only and (B, D) TF-only events with high accuracies. Computational models were trained and applied to the same SP1-TF pair in the same cell line, indicated by colors as legend: black, A549; red, GM12878; cyan, H1-hESC; green, HepG-2; and blue, K562. Different chromatin features were included in each test: (A, B) the combinations of all or a few features, and (C, D) individual HMs.**
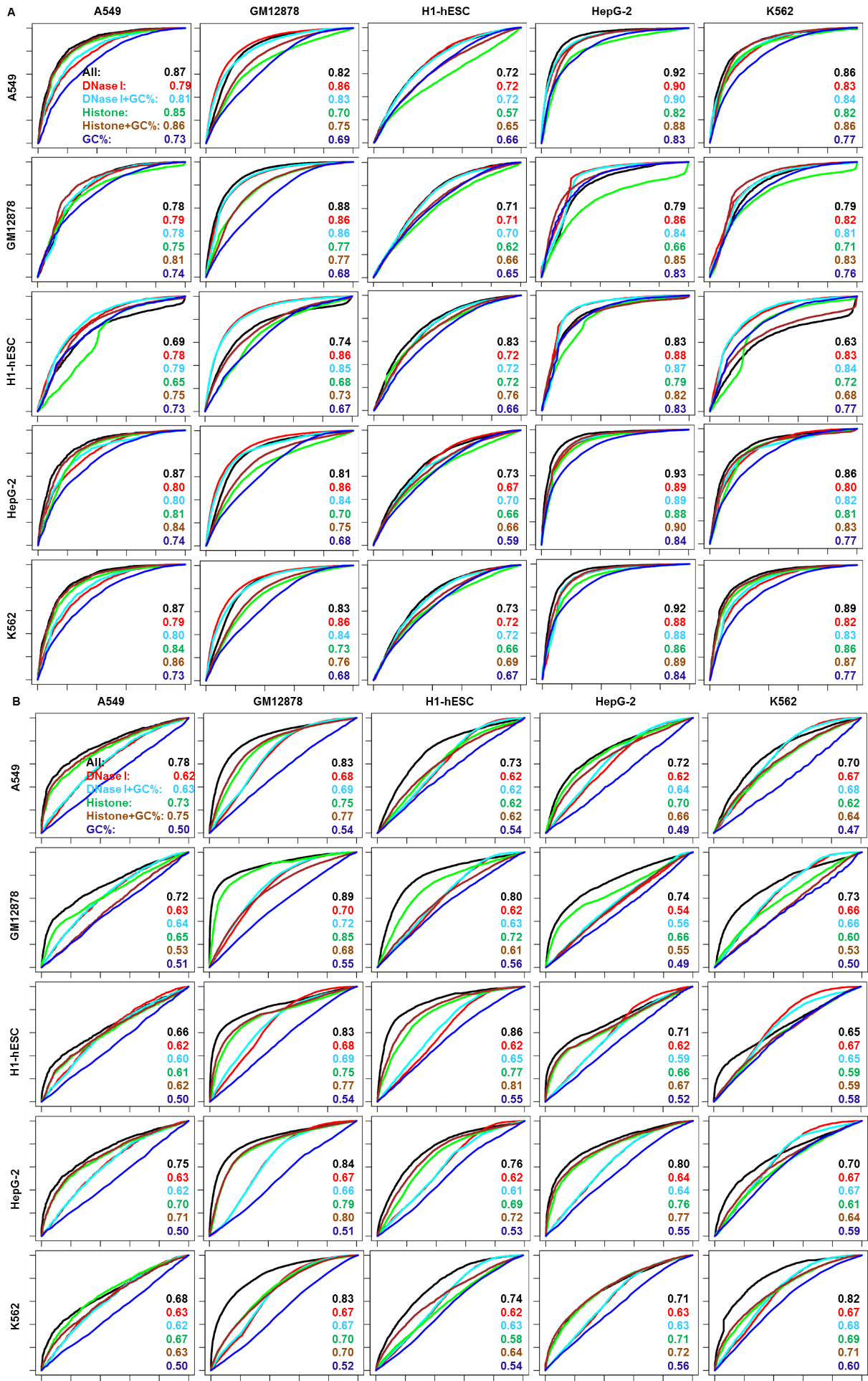
**

**Supplemental Figure S15:** Chromatin features enable predictions of CTCF-TF co-occupancy across cell lines. Shown are ROC curves with colors representing the predictions using different chromatin features. Models were trained in the cell line indicated by the row and tested on each of the five cell lines indicated by the column. The AUC values are indicated on the plot as legend.

**
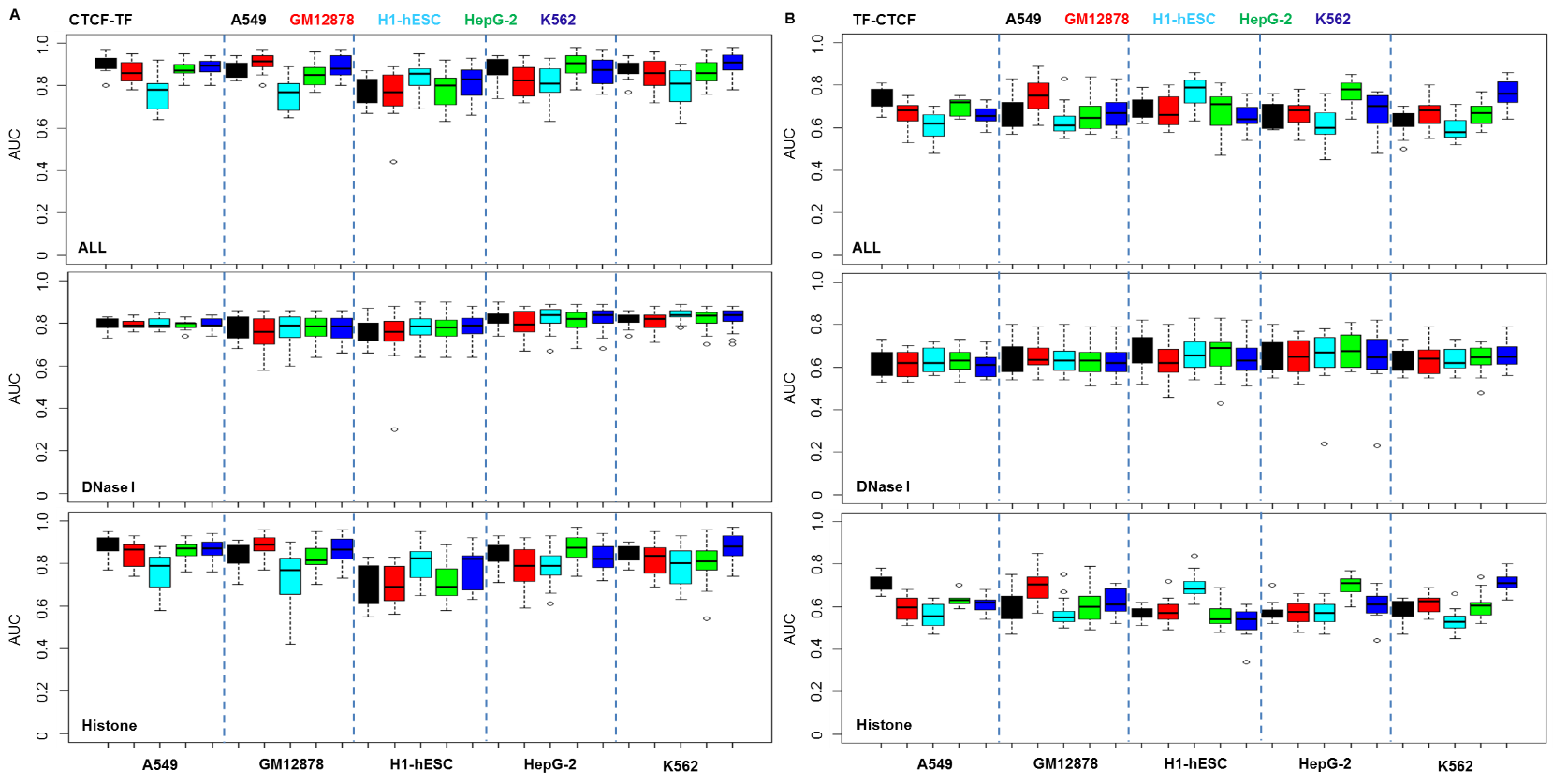
**

**Supplemental Figure S16:** Chromatin features enable the predictions of (A) CTCF-TF co-occupancy from CTCF-only, and (B) TF-CTCF co-occupancy from TF-only events across cell lines. Average prediction accuracies were shown when models from one cell line, indicated by color legend, were applied to another cell, given by x-axis. All features were used in this test. Different features were considered in each test.

**
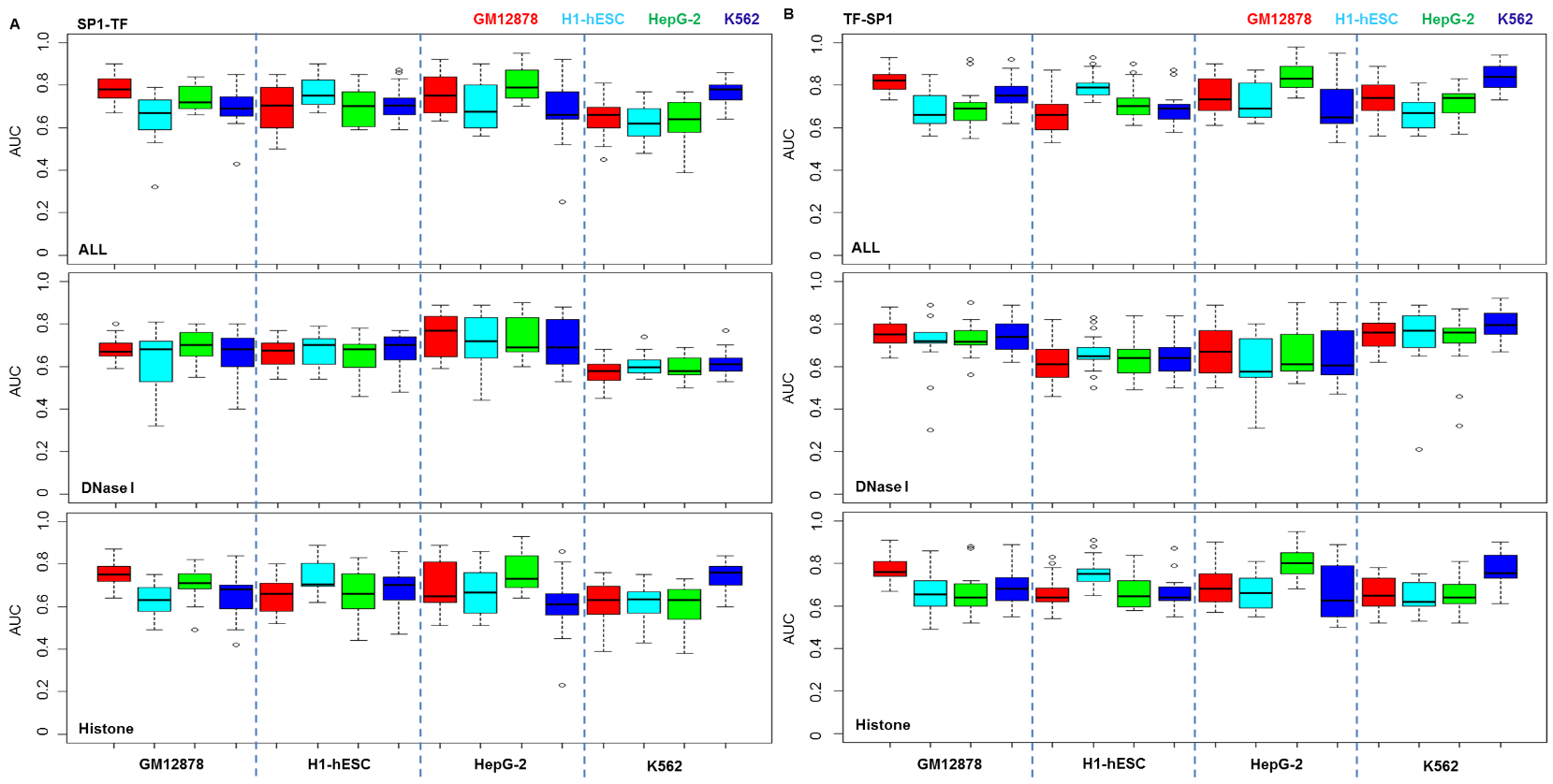
**

**Supplemental Figure S17:** Chromatin features enable the predictions of (A) SP1-TF co-occupancy from SP1-only, and (B) TF-SP1 co-occupancy from TF-only events across cell lines. Average prediction accuracies were shown when models from one cell line, indicated by color legend, were applied to another cell, given by x-axis. All features were used in this test. Different features were considered in each test.

**
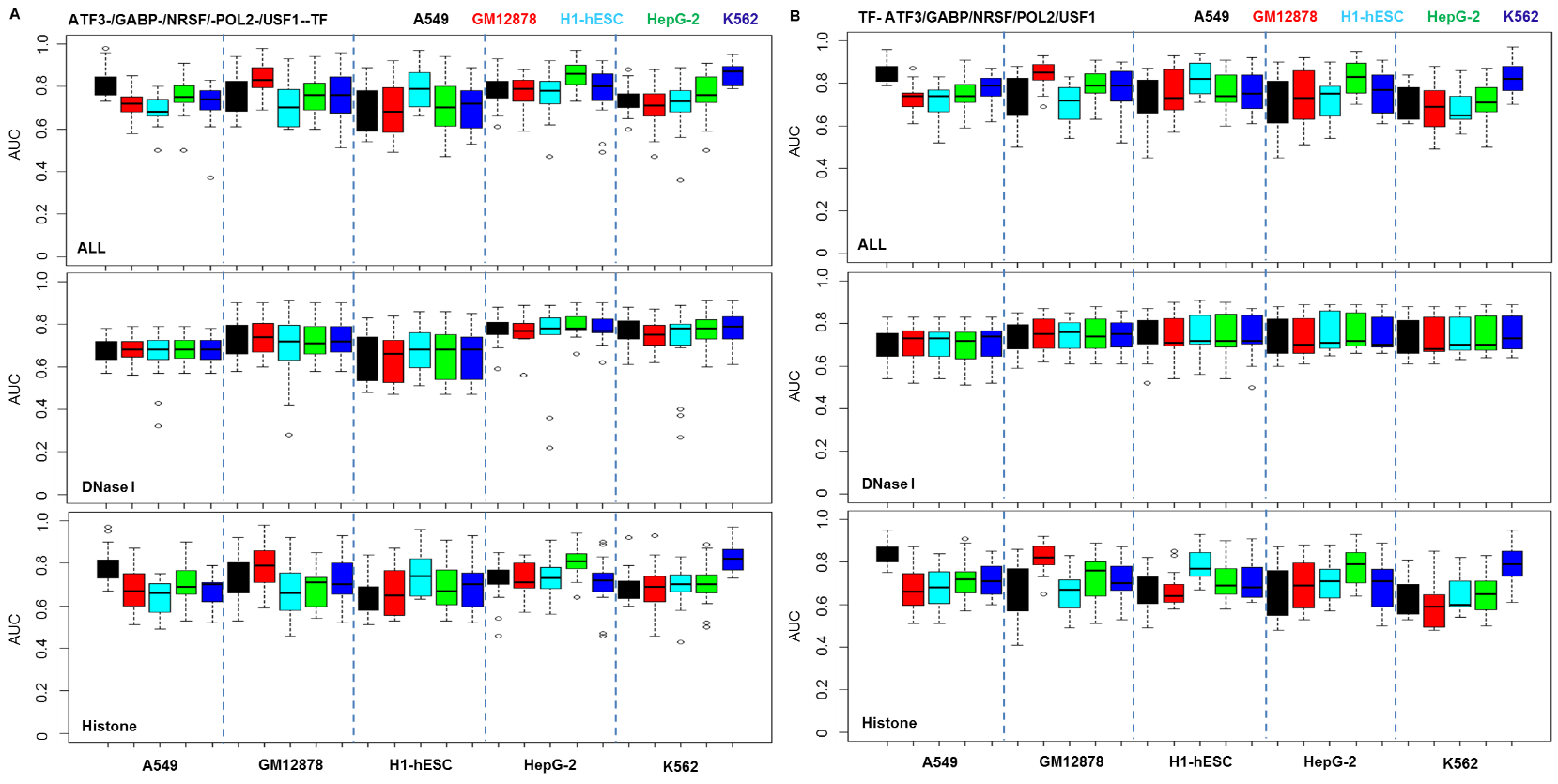
**

**Supplemental Figure S18:** Chromatin features enable the predictions of (A) ATF3-/GABP-/NRSF-/POL2-/USF1-TF co-occupancy from ATF3-/GABP-/NRSF/-POL2-/USF1-only, and (B) TF-ATF3/GABP/NRSF/POL2-/USF1 co-occupancy from TF-only events across cell lines. Average prediction accuracies were shown when models from one cell line, indicated by color legend, were applied to another cell, given by x-axis. All features were used in this test. Different features were considered in each test.

**
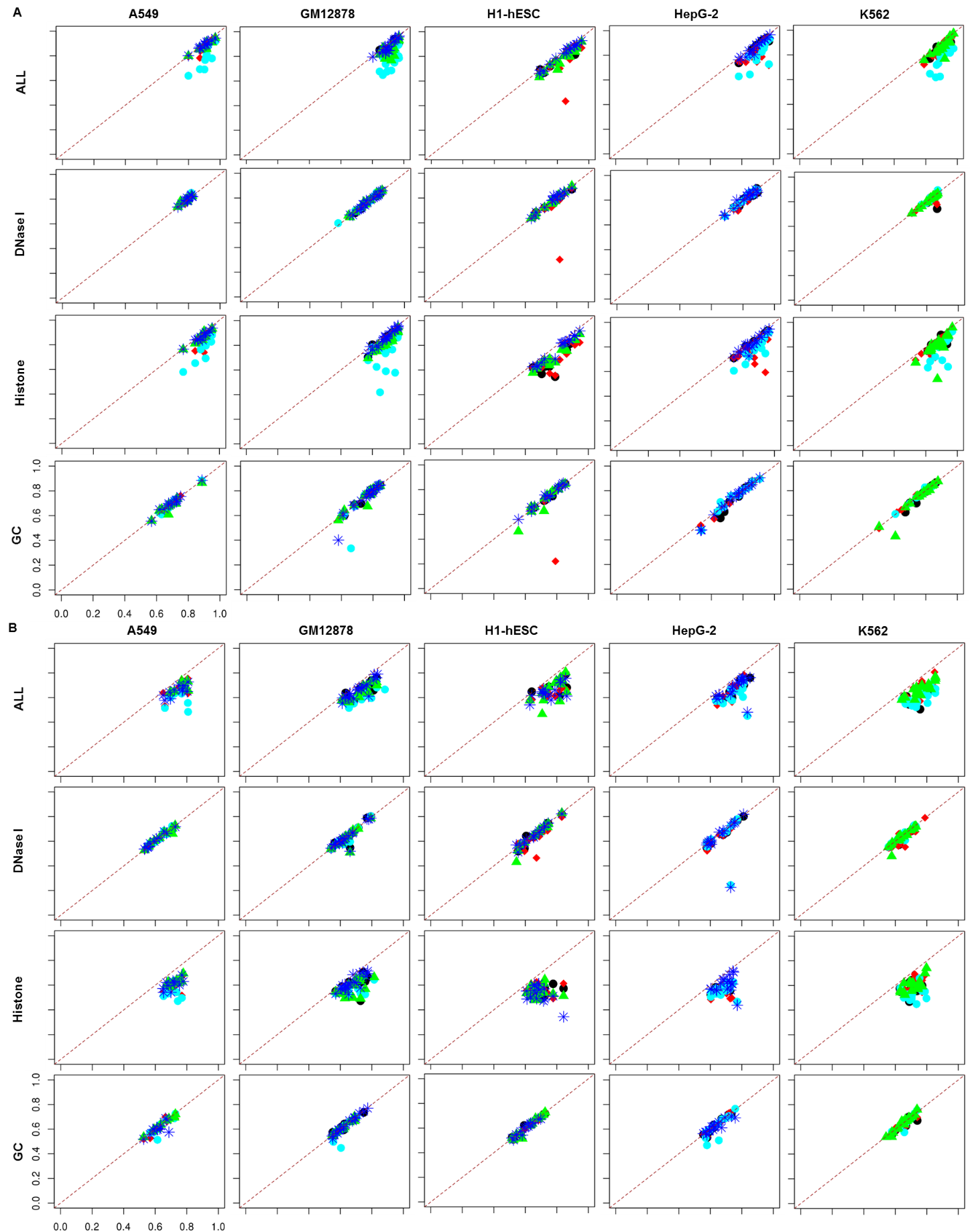
**

**Supplemental Figure S19:** Comparisons of prediction accuracy (AUC) of (A) CTCF-TF and (B) TF-CTCF co-occurrence. Comparisons were performed between predictions using models obtained and applied in the same cell line (column name, AUC is given by y-axis) and predictions using models obtained from other cell lines (x-axis) represented by colored points: black: A549, red: GM12878, cyan: H1-hESC, green: HepG-2, and blue: K562. Different chromatin features were included when training the model indicated by row names.

**
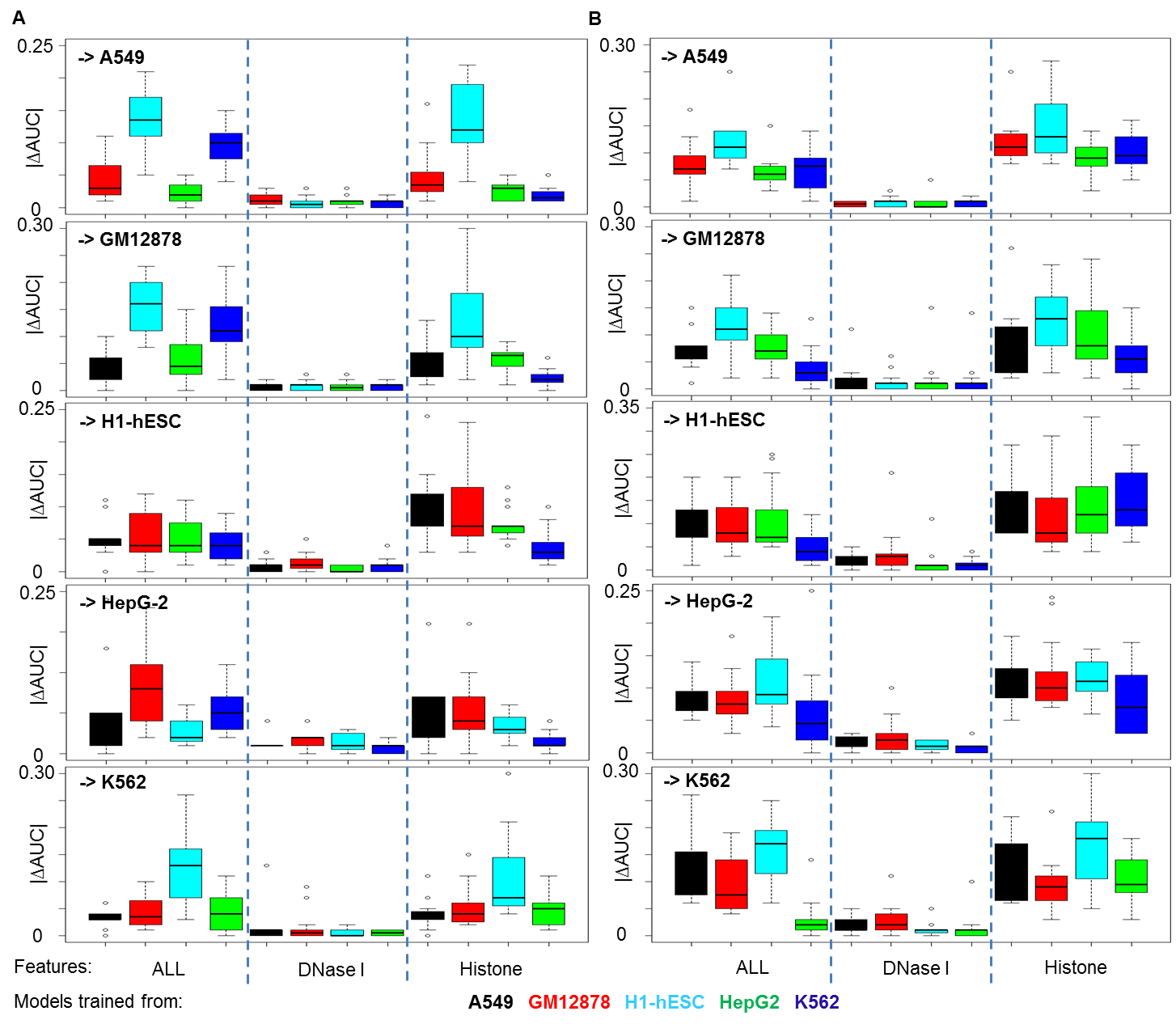
**

**Supplemental Figure S20:** Boxplots of difference of prediction accuracies (|ΔAUC|) for (A) CTCF-TF and (B) TF-CTCF co-occurrence. Models with different features, indicated by x-axis, were trained in other cell types, indicated by colors, and then applied to the test cell line. |ΔAUC| was calculated by subtracting cross cell predictions from predictions with model trained and applied to the same cell line**.**

**
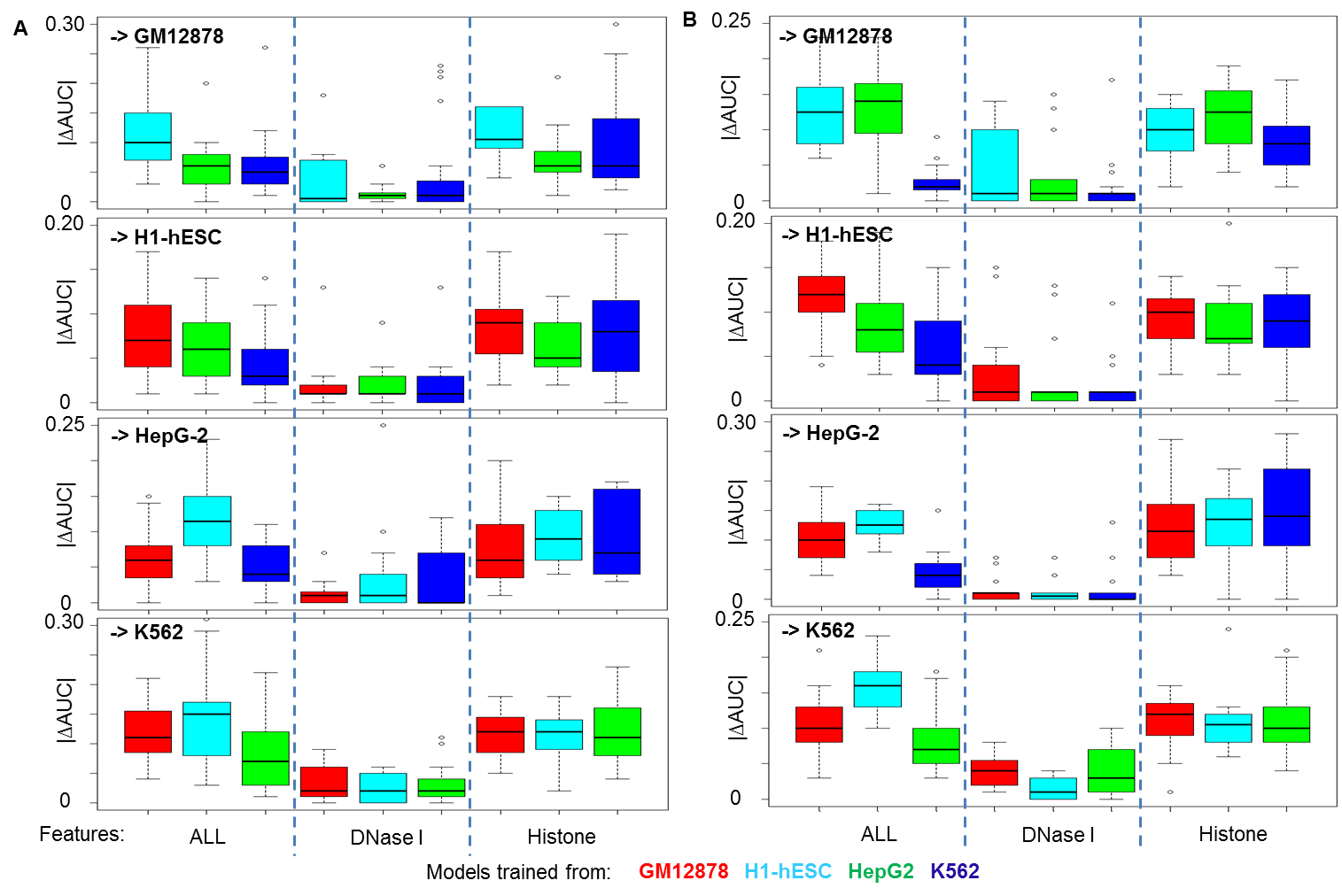
**

**Supplemental Figure S21:** Boxplots of difference of prediction accuracies (|ΔAUC|) for (A) SP1-TF and (B) TF-SP1 co-occurrence. Models with different features, indicated by x-axis, were trained in other cell types, indicated by colors, and then applied to the test cell line. |ΔAUC| was calculated by subtracting cross cell predictions from predictions with model trained and applied to the same cell line**.**

**
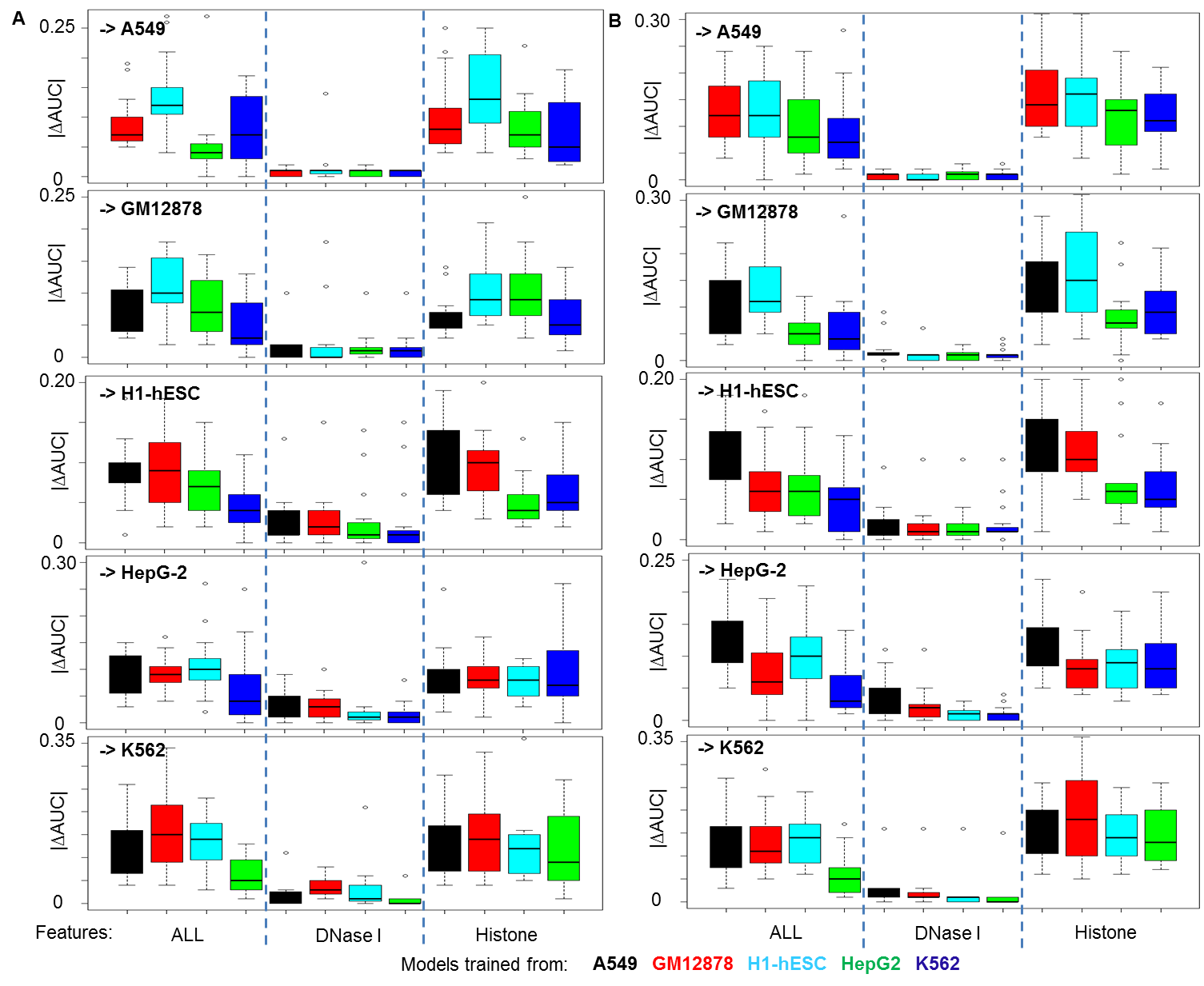
**

**Supplemental Figure S22:** Boxplots of difference of prediction accuracies (|ΔAUC|) for (A) ATF3-/GABP-/NRSF-/POL2-/USF1-TF and (B) TF-ATF3/GABP/NRSF/POL2/USF1 co-occurrence. Models with different features, indicated by x-axis, were trained in other cell types, indicated by colors, and then applied to the test cell line. |ΔAUC| was calculated by subtracting cross cell predictions from predictions with model trained and applied to the same cell line**.**

**
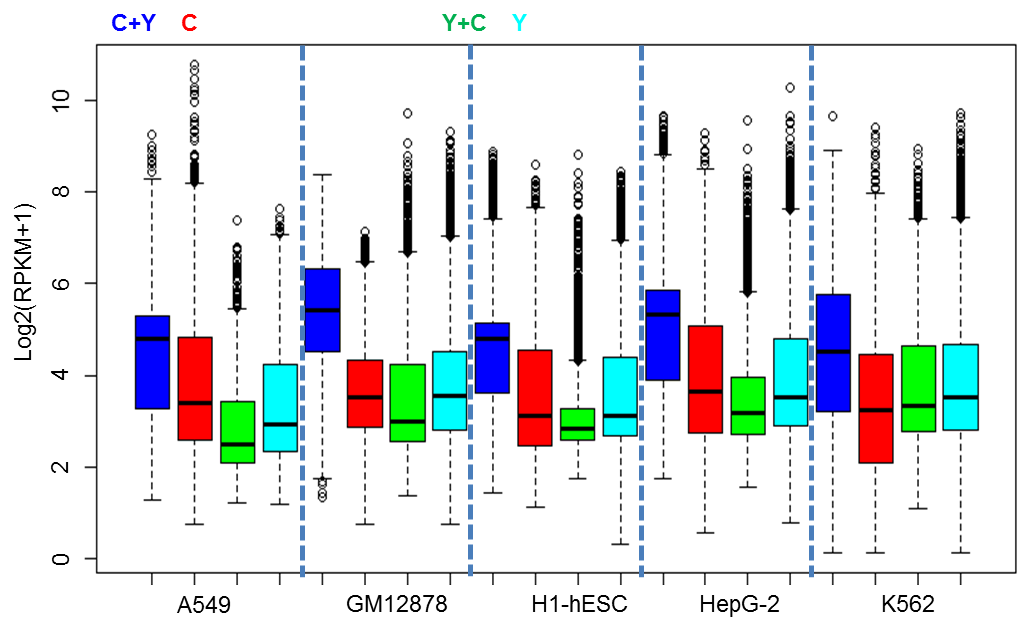
**

**Supplemental Figure S23:** (A) Comparison of binding affinities of CTCF locating at CTCF-YY1 co-occupying (C+Y, blue) and CTCF-only binding (C, red) sites, as well as binding affinities of YY1 locating at YY1-CTCF co- occupying (Y+C, green) and YY1-only events (Y, cyan). Student’s *t* test was performed with each comparing pair. *p* value is < 1.0E-16 unless indicated in the figure. (B) Distributions of CTCF/YY1 binding sites across different binding events in different genomic regions.

**
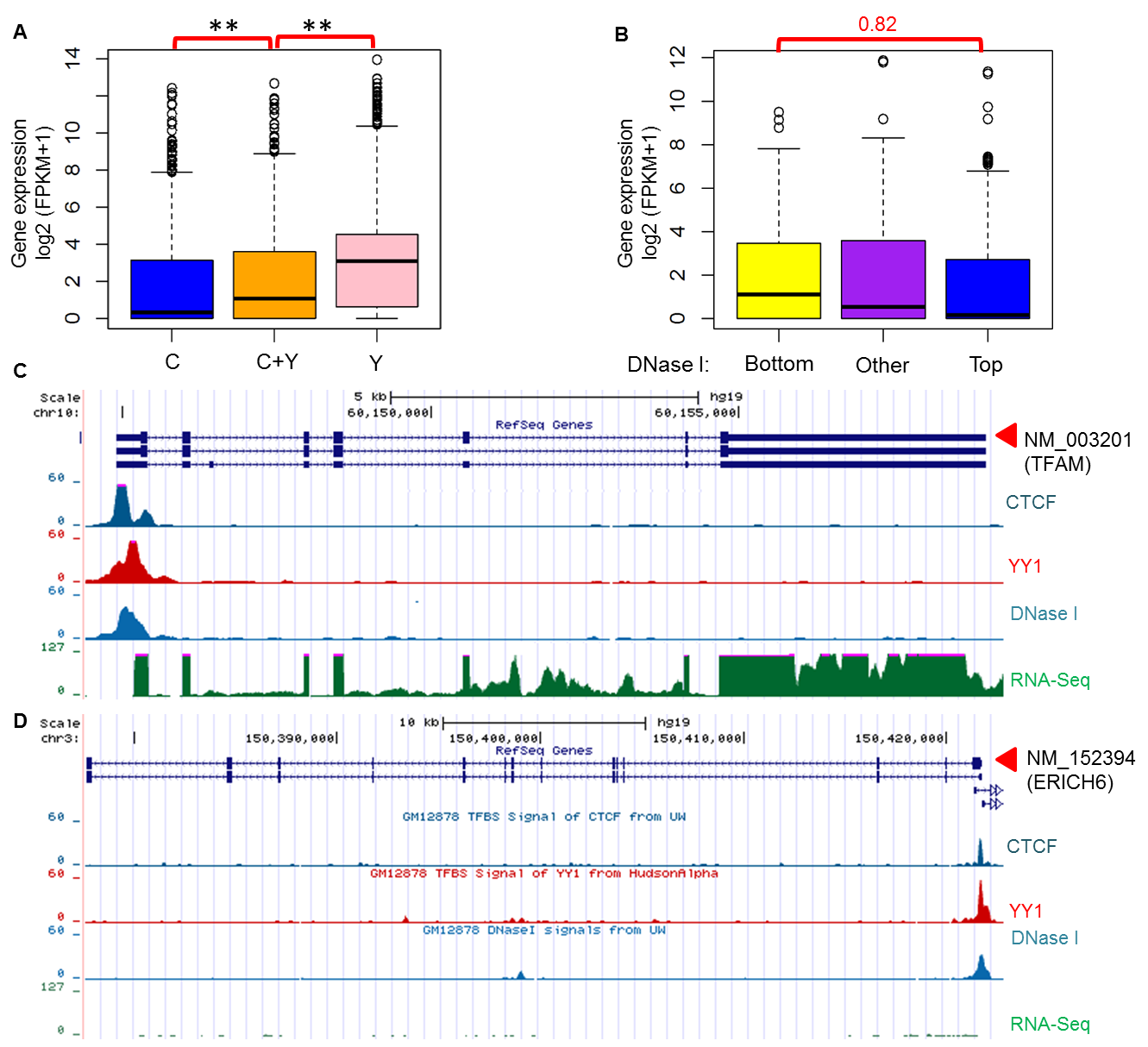
**

**Supplemental Figure S24:** Association of CTCF-YY1 co-occupancy, DNase I and transcript activity. (A) Expression level of RefSeq genes overlapping CTCF-only, YY1-only and CTCF-YY1 binding events in the GM12878 cell line. ** p < 0.01, Wilcoxon rank-sum test, C versus C+Y and Y versus C+Y. (B) Expression level of RefSeq genes with at least one CTCF-YY1 binding events but no CTCF-only or YY1-only binding events. Genes were ranked according to the density of DNase I signal overlapped with CTCF-YY1 binding regions, and categorized into the Top quantile, Bottom quantile, and Other quantiles of DNase I signal. (C and D) CTCF, YY1, DNase I and RNA-Seq profiles of genes. Both genes have CTCF-YY1 binding and DNase I signal in their promoters. But the gene expression level is high for NM_003201 (TFAM) (C) and low for NM_152394 (ERICH6) (D).

**
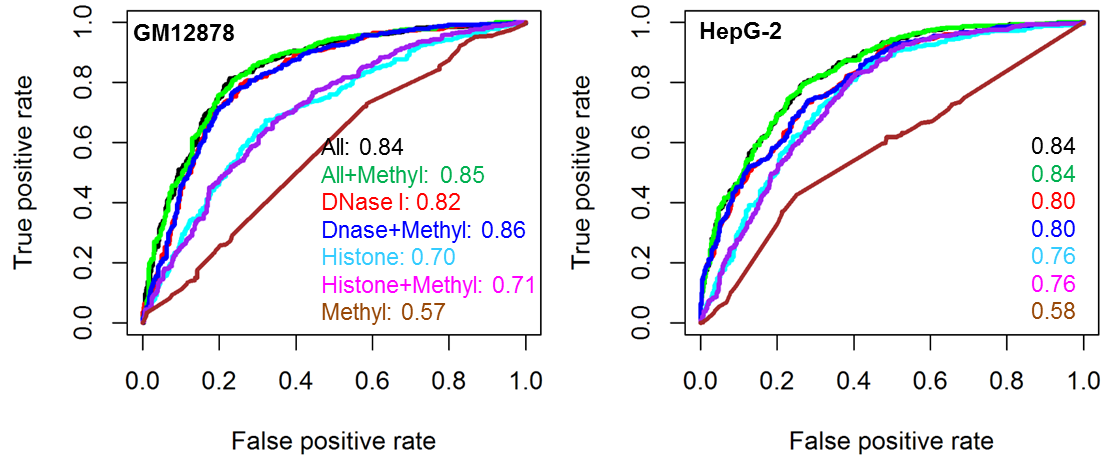
**

**Supplemental Figure S25:** Chromatin features, including DNA methylation, are predictive of CTCF-YY1 co-occupancy in the GM12878 and HepG-2 cell lines. ROC curves are shown with colors representing predictions with different chromatin features, and AUC values are indicated in the legend. We note that “All” includes DNase I, GC-content and 11 HMs.


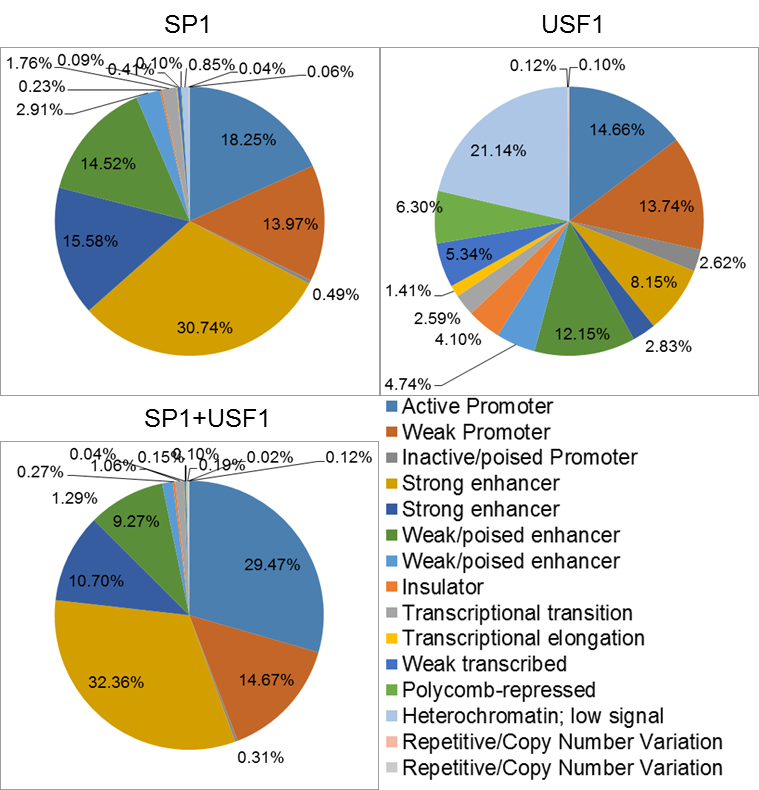


**Supplemental Figure S26:** Distributions of SP1/USF1 binding sites (in the HepG-2 cell line) across different binding events in different genomic regions annotated with 15 distinct chromatin states. These 15 states were defined by the ChromHMM using nine factors, including CTCF binding, H3K27ac, H3K27me3, H3K36me3, H3K4me1, H3K4me2, H3K4me3, H3K9ac, and H4K20me1, and input.


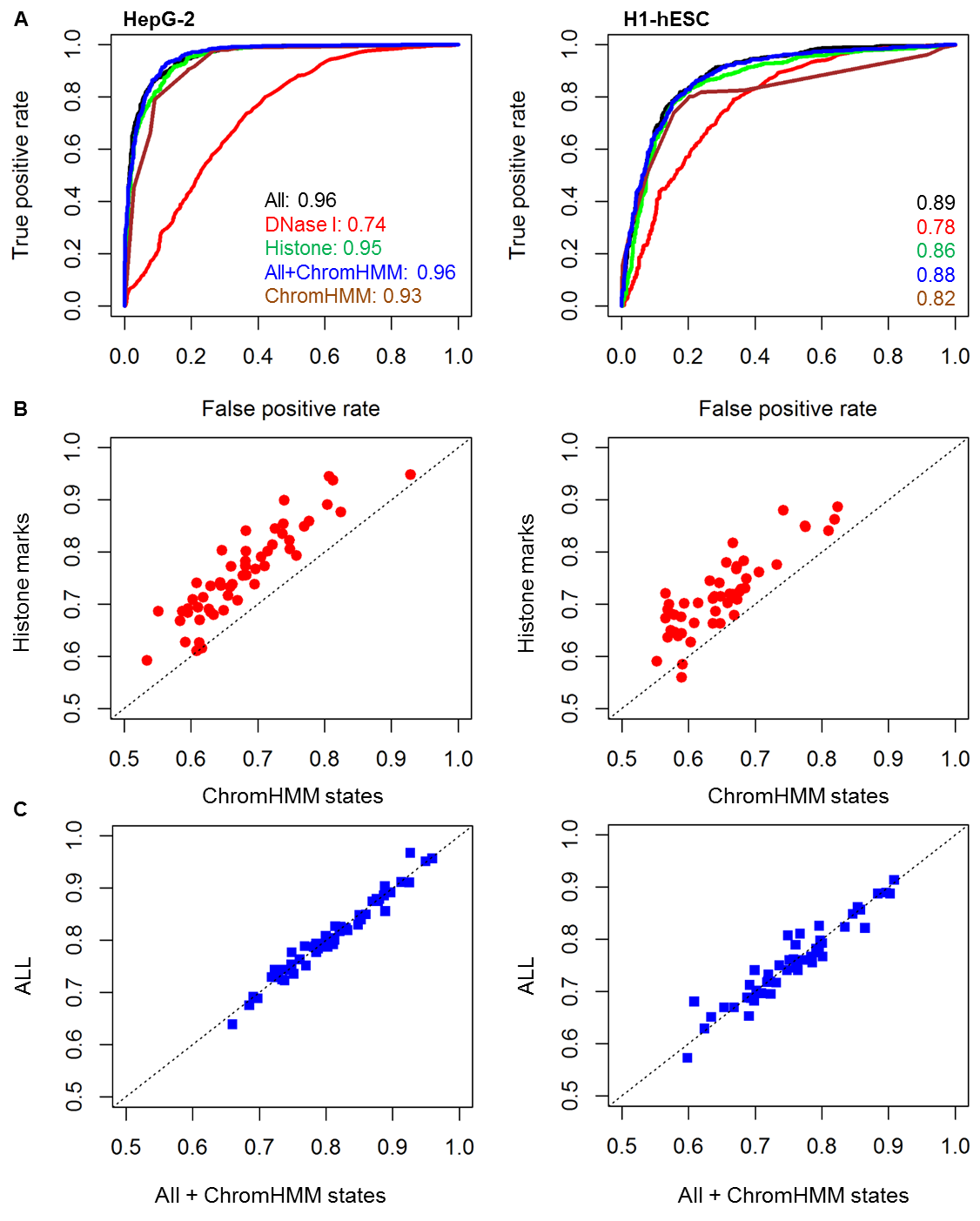


**Supplemental Figure S27:** Chromatin features, including chromatin state segmentation, are predictive of TF-TF co-occupancy in the HepG-2 and H1-hESC cell lines. (A) Prediction of USF1-SP1 co-occupancy with different features. ROC curves are shown with the corresponding colors representing predictions with different chromatin features, and AUC values are indicated in the legend. We note that “All” includes DNase I, GC-content and 11 HMs. (B) Comparisons of predictive ability of histone marks and chromatin state segmentation defined with the ChromHMM. (C) Comparisons of prediction accuracy between models using all chromatin features, including 11 HMs, DNase I and GC component, and models using all chromatin features and chromatin segmentation states.

**REFERENCE**

1. Landt, S.G., Marinov, G.K., Kundaje, A., Kheradpour, P., Pauli, F., Batzoglou, S., Bernstein, B.E., Bickel, P., Brown, J.B., Cayting, P. *et al.* (2012) ChIP-seq guidelines and practices of the ENCODE and modENCODE consortia. *Genome Res*, **22**, 1813-1831.

2. Bailey, T.L. and Elkan, C. (1994) Fitting a mixture model by expectation maximization to discover motifs in biopolymers. *Proc Int Conf Intell Syst Mol Biol*, **2**, 28-36.

3. Schwalie, P.C., Ward, M.C., Cain, C.E., Faure, A.J., Gilad, Y., Odom, D.T. and Flicek, P. (2013) Co-binding by YY1 identifies the transcriptionally active, highly conserved set of CTCF-bound regions in primate genomes. *Genome Biol*, **14**, R148.

4. Fenouil, R., Cauchy, P., Koch, F., Descostes, N., Cabeza, J.Z., Innocenti, C., Ferrier, P., Spicuglia, S., Gut, M., Gut, I. *et al.* (2012) CpG islands and GC content dictate nucleosome depletion in a transcription-independent manner at mammalian promoters. *Genome Res*, **22**, 2399-2408.

5. Wang, J., Zhuang, J., Iyer, S., Lin, X., Whitfield, T.W., Greven, M.C., Pierce, B.G., Dong, X., Kundaje, A., Cheng, Y. *et al.* (2012) Sequence features and chromatin structure around the genomic regions bound by 119 human transcription factors. *Genome Res*, **22**, 1798-1812.

6. Kudla, G., Lipinski, L., Caffin, F., Helwak, A. and Zylicz, M. (2006) High guanine and cytosine content increases mRNA levels in mammalian cells. *PLoS Biol*, **4**, e180.

7. Ernst, J. and Kellis, M. (2012) ChromHMM: automating chromatin-state discovery and characterization. *Nat Methods*, **9**, 215-216.

8. Bernstein, B.E., Meissner, A. and Lander, E.S. (2007) The mammalian epigenome. *Cell*, **128**, 669-681.

9. Berger, S.L. (2007) The complex language of chromatin regulation during transcription. *Nature*, **447**, 407-412.

10. Mikkelsen, T.S., Ku, M., Jaffe, D.B., Issac, B., Lieberman, E., Giannoukos, G., Alvarez, P., Brockman, W., Kim, T.K., Koche, R.P. *et al.* (2007) Genome-wide maps of chromatin state in pluripotent and lineage-committed cells. *Nature*, **448**, 553-560.

11. Young, M.D., Willson, T.A., Wakefield, M.J., Trounson, E., Hilton, D.J., Blewitt, M.E., Oshlack, A. and Majewski, I.J. (2011) ChIP-seq analysis reveals distinct H3K27me3 profiles that correlate with transcriptional activity. *Nucleic Acids Res*, **39**, 7415-7427.

12. Heintzman, N.D., Stuart, R.K., Hon, G., Fu, Y., Ching, C.W., Hawkins, R.D., Barrera, L.O., Van Calcar, S., Qu, C., Ching, K.A. *et al.* (2007) Distinct and predictive chromatin signatures of transcriptional promoters and enhancers in the human genome. *Nat Genet*, **39**, 311-318.

13. Zhang, X., Bernatavichute, Y.V., Cokus, S., Pellegrini, M. and Jacobsen, S.E. (2009) Genome-wide analysis of mono-, di- and trimethylation of histone H3 lysine 4 in Arabidopsis thaliana. *Genome Biol*, **10**, R62.

14. Roadmap Epigenomics, C., Kundaje, A., Meuleman, W., Ernst, J., Bilenky, M., Yen, A., Heravi-Moussavi, A., Kheradpour, P., Zhang, Z., Wang, J. *et al.* (2015) Integrative analysis of 111 reference human epigenomes. *Nature*, **518**, 317-330.

15. Thurman, R.E., Rynes, E., Humbert, R., Vierstra, J., Maurano, M.T., Haugen, E., Sheffield, N.C., Stergachis, A.B., Wang, H., Vernot, B. *et al.* (2012) The accessible chromatin landscape of the human genome. *Nature*, **489**, 75-82.

16. Chen, H., Li, H., Liu, F., Zheng, X., Wang, S., Bo, X. and Shu, W. (2015) An integrative analysis of TFBS-clustered regions reveals new transcriptional regulation models on the accessible chromatin landscape. *Sci Rep*, **5**, 8465.

17. Arvey, A., Agius, P., Noble, W.S. and Leslie, C. (2012) Sequence and chromatin determinants of cell-type-specific transcription factor binding. *Genome Res*, **22**, 1723-1734.

18. He, H.H., Meyer, C.A., Hu, S.S., Chen, M.W., Zang, C., Liu, Y., Rao, P.K., Fei, T., Xu, H., Long, H. *et al.* (2014) Refined DNase-seq protocol and data analysis reveals intrinsic bias in transcription factor footprint identification. *Nat Methods*, **11**, 73-78.
